# The biological process of lysine‐tRNA charging is therapeutically targetable in liver cancer

**DOI:** 10.1111/liv.14692

**Published:** 2020-10-20

**Authors:** Ruyi Zhang, Lisanne Noordam, Xumin Ou, Buyun Ma, Yunlong Li, Pronay Das, Shaojun Shi, Jiaye Liu, Ling Wang, Pengfei Li, Monique M. A. Verstegen, D. Srinivasa Reddy, Luc J. W. van der Laan, Maikel P. Peppelenbosch, Jaap Kwekkeboom, Ron Smits, Qiuwei Pan

**Affiliations:** ^1^ Department of Gastroenterology and Hepatology Erasmus MC‐University Medical Center Rotterdam The Netherlands; ^2^ Institute of Preventive Veterinary Medicine Sichuan Agricultural University Chengdu China; ^3^ Organic Chemistry Division CSIR‐National Chemical Laboratory Pune India; ^4^ Department of Surgery Erasmus MC‐University Medical Center Rotterdam The Netherlands

**Keywords:** cladosporin, liver cancer, lysine, Lysyl‐tRNA Synthetase, tRNA‐Lys‐CUU, tRNAome

## Abstract

**Background & Aims:**

Mature transfer RNAs (tRNA) charged with amino acids decode mRNA to synthesize proteins. Dysregulation of translational machineries has a fundamental impact on cancer biology. This study aims to map the tRNAome landscape in liver cancer patients and to explore potential therapeutic targets at the interface of charging amino acid with tRNA.

**Methods:**

Resected tumour and paired tumour‐free (TFL) tissues from hepatocellular carcinoma (HCC) patients (n = 69), and healthy liver tissues from organ transplant donors (n = 21), HCC cell lines, and cholangiocarcinoma (CC) patient‐derived tumour organoids were used.

**Results:**

The expression levels of different mature tRNAs were highly correlated and closely clustered within individual tissues, suggesting that different members of the tRNAome function cooperatively in protein translation. Interestingly, high expression of tRNA‐Lys‐CUU in HCC tumours was associated with more tumour recurrence (HR 1.1; *P* = .022) and worse patient survival (HR 1.1; *P* = .0037). The expression of Lysyl‐tRNA Synthetase (KARS), the enzyme catalysing the charge of lysine to tRNA‐Lys‐CUU, was significantly upregulated in HCC tumour tissues compared to tumour‐free liver tissues. In HCC cell lines, lysine deprivation, KARS knockdown or treatment with the KARS inhibitor cladosporin effectively inhibited overall cell growth, single cell‐based colony formation and cell migration. This was mechanistically mediated by cell cycling arrest and induction of apoptosis. Finally, these inhibitory effects were confirmed in 3D cultured patient‐derived CC organoids.

**Conclusions:**

The biological process of charging tRNA‐Lys‐CUU with lysine sustains liver cancer cell growth and migration, and is clinically relevant in HCC patients. This process can be therapeutically targeted and represents an unexplored territory for developing novel treatment strategies against liver cancer.

**Abbreviations**
ARSs: Aminoacyl‐tRNA synthetases
CC: cholangiocarcinoma
HCC: hepatocellular carcinoma
KARS: Lysyl‐tRNA Synthetase
TFL: tumour‐free liver
tRNA: transfer RNA

**Lay summary**

Dysregulation of the protein synthesis machinery in cancer has a major impact on pathophysiology, including cancer development and progression. This study found that the biological process of charging lysine to the corresponding tRNA is altered in liver cancer patients. This in turn can be explored for developing potential therapeutic strategies to treat liver cancer.

## 1 INTRODUCTION

Translation, the synthesis of protein from mRNA, is a fundamental biological process required for virtually all cellular functions.^1^ A key step in protein synthesis is the recognition of codons by the transfer RNAs (tRNA) charged with their corresponding amino acids.^2^, ^3^ tRNAs are transcribed from genomic DNA, have a length of typically 70‐90 base pairs and are subjected to post‐transcriptional modification. The addition of a common CCA ribonucleotide sequence to the 3′ end of tRNA enables charging with an amino acid. Aminoacyl‐tRNA synthetases (ARSs) catalyse the charging of amino acids to their cognate mature tRNAs, thereby providing the substrates for global protein synthesis.^4^ Theoretically, 61 types of aminoacyl‐tRNAs would be required for decoding the 61 triplet codons that specify 20 amino acids. However, the minimally required tRNA species for decoding in real life is usually less than the theoretically calculated number.^5^ From the GtRNA 2.0 database, we have recently retrieved 57 human mature tRNA species constituting as the tRNAome.^6^

Dysregulation of gene translation has been well‐recognized in cancer development and progression.^7^, ^8^ Involvement of tRNAs has been demonstrated in breast cancer, lung cancer and melanoma.^9^ However, despite the biological importance of tRNA, it has hardly been investigated in depth, which includes the setting of cancer research. One of the main challenges is that tRNAs are technically difficult to be quantified because of redundancy in genomic copies, extremely short sequences, rigid secondary structure and post‐transcriptional modifications. A recent study has analysed the global expression landscape of tRNAs across 31 cancer types. They used the Cancer Genome Atlas database and analysed tRNA profiles from miRNA‐sequencing data, as an indirect interpretation of tRNA expression.^10^ However, the functional forms are the mature tRNAs charged with amino acids. Based on these unique characteristics, we have developed a simplified qRT‐PCR assay to quantify the mature tRNAome.^6^

Several enzymes participate in the modification and maturation of tRNAs. ARSs responsible for charging amino acids have evolutionarily conserved enzymatic properties. As protein synthesis is universally accelerated in proliferating malignant cells, the expression levels of many ARSs are increased.^11^ In contrast with tRNAs, the functions of ARSs have been widely investigated and various therapeutic approaches have been explored, including the development of pharmacological inhibitors targeting the catalytic sites.^12^ This has provided new avenues for identifying unexplored therapeutic targets and developing effective anti‐cancer therapeutics.

Primary liver cancer, mainly consisting of hepatocellular carcinoma (HCC) and cholangiocarcinoma (CC), is among the leading causes of cancer‐related deaths with limited treatment options available. In this study, we aim to profile the tRNAome landscape in tumours of HCC patients using our recently developed mature tRNA quantification assay.^6^ Secondly, we aim to assess the therapeutic potential of targeting the interface of amino acid charging to tRNA in experimental models of liver cancer.

## 2 PATIENTS, MATERIALS AND METHODS

### 2.1 Patient tissues and information

Fresh frozen tumour tissues and paired tumour‐free liver (TFL) tissues, located at least 2 cm from the tumour, were collected after surgery or retrieved from the archives of the Department of Pathology, Erasmus Medical Center Rotterdam. The included patients underwent hepatic resection (n = 68) or liver transplantation (n = 1) for HCC at the Erasmus Medical Center between February 1995 and September 2017. Diagnosis of HCC was confirmed by histopathological examination and medical records were reviewed for clinicopathological characteristics including date of first recurrence, HCC‐specific death, liver transplantation (if applicable) and last follow‐up (Table 1; Table S1). Tissue samples (n = 21) of healthy livers from organ donors that were used for liver transplantation were used as healthy controls. This study was approved by the Erasmus MC Medical Ethical Committee and adhered to the 1975 Declaration of Helsinki.

**Table 1:** Patient characteristics

Characteristics	HCC patients (n = 69)
Age at treatment (years)	
Mean ± SD	60 ± 16
Median (range)	64 (11‐82)
Sex, no. (%)	
Male	41 (59.4)
Female	28 (40.6)
Race, no (%)	
White	58 (84.1)
African	6 (8.7)
Asian	4 (5.8)
Not reported	1 (1.4)
Etiology, no (%)	
No known liver disease	20 (29.0)
Alcohol	16 (23.2)
NASH	8 (11.6)
Hepatitis B	8 (11.6)
Hepatitis C + Alcohol	5 (7.2)
Hepatitis B + Alc/HepC/HepD/NASH	4 (5.8)
Hepatitis C	4 (5.8)
Fibrolamellar HCC	3 (4.4)
Other	1 (1.5)
Hepatitis status, no (%)	
Hepatitis B or C positive	21 (30.4)
Chronic Hepatitis B	12 (17.4)
Chronic Hepatitis C	10 (14.5)
Cirrhosis, no (%)	
Yes	50 (72.5)
No	19 (27.5)
Differentiation grade, no (%)	
Good	8 (11.6)

### 2.2 Reagents

The natural product cladosporin was synthesized at the CSIR‐National Chemical Laboratory, India, and the NMR data of cladosporin are shown in Figure S1. It was dissolved in dimethyl sulphoxide (DMSO) at a stock concentration of 100 mM. L‐Arginine, N‐acetylcysteine, gastrin, nicotinamide and 3‐(4,5‐dimethylthiazol‐2‐yl)‐2,5‐diphenyltetrazolium bromide (MTT) were purchased from Sigma‐Aldrich. Dulbecco's modified Eagle's medium (DMEM, #41965039) and DMEM for SILAC (#88364) were purchased from Life Technologies Europe BV. Advanced DMEM/F‐12 (#12634‐010) and SILAC Advanced DMEM/F‐12 Flex Media, no L‐Lysine, no L‐Arginine, no glucose, no phenol red (#A2494301) were purchased from Gibco Life Technologies. L‐Lysine was purchased from Bio‐Connect BV. Matrigel was purchased from BD Bioscience. Cytokines, B27 and N2 were purchased from Invitrogen. EGF, FGF10 and HGF were purchased from Peprotech Company.

### 2.3 Cell culture and amino acid deprivation

HCC cell lines, including Huh6, Huh7, PLC/PRF/5, SNU398, SNU449, SNU182, Hep3B, HepG2 and human embryonic kidney epithelial cell line 293T (HEK293T) were grown in DMEM (GIBCO Life Technologies), supplemented with 10% (v/v) fetal bovine serum (Hyclone Technologies), 100 units/mL of penicillin and 100 µg/mL of streptomycin. To establish a lysine deprivation model, L‐arginine (0.4 mM; the same concentration as in DMEM) was firstly added to “DMEM for SILAC” (L‐lysine and L‐arginine deficiency) medium, and then different concentrations of L‐lysine were added. All the cells were incubated at 37°C in a humidified atmosphere containing 5% CO_2_. Authentication of all the cell lines was performed using the short tandem repeat genotyping assay at the Department of Pathology, Erasmus Medical Center Rotterdam. The mycoplasma‐free status was regularly commercially checked and confirmed by GATC Biotech Konstanz, Germany.

To further test the deprivation of each of the nine essential amino acids, culture medium was prepared with Earle Balanced Salt Solution (H9269; Sigma‐Aldrich), Glucose and MEM vitamin solution (100×; M6895; Sigma‐Aldrich). Different amino acids except for the deprived one were then added, according to the concentrations of amino acids in regular complete DMEM medium (#41965; Gibco).^13^, ^14^

### 2.4 Organoid culture and lysine deprivation

The establishment and maintenance of human normal liver and liver tumour organoids were performed as described.^15^ Three batches of normal liver organoids (HLO‐1, HLO‐2, HLO‐3) were derived from intrahepatic biliary epithelial progenitor cells. Three batches of liver tumour organoids (CCO‐1, CCO‐2, CCO‐3) were derived from tumour tissue of CC patients. The use of human organoids was approved by the Erasmus MC Medical Ethical Committee. To establish a lysine deprivation model, L‐Arginine (0.7 mM; the same concentration as in Advanced DMEM/F‐12 DMEM) was firstly added to “Advanced DMEM/F‐12 without L‐Lysine, L‐Arginine and no‐phenol red” medium, and then different concentrations of L‐Lysine were added. The culture medium for human normal liver organoids and liver tumour organoids was basic medium (Advanced/F12 DMEM with 1% penicillin/streptomycin, 1% L‐Glutamine and 10 mM HEPES) supplemented with 1:50 B27 supplement (without vitamin A), 1:100 N2 supplement, 1 mM N‐acetylcysteine, 10% (vol/vol) R‐spondin 1 (conditioned medium), 10 mM nicotinamide, 10 nM recombinant human [Leu^15^]‐gastrin I, 50 ng/mL recombinant human EGF, 100 ng/mL recombinant human FGF10, 25 ng/mL recombinant human HGF, 10 µM Forskolin and 5 µM A83‐01. The medium was replaced every 3 days and passage was performed according to the growth of organoids.

### 2.5 Lentiviral shRNA packaging and transduction

To perform gene knockdown, pLKO.1‐based lentiviral vectors (Sigma‐Aldrich) targeting KARS and a scrambled control vector (shCTR) were obtained from the Erasmus Medical Center for Biomics. Lentiviral pseudoparticles were generated in HEK293T cells. After 2 days of transfection with the lentiviral particles, Huh7 and SNU398 cells were subsequently selected by 3 µg/mL puromycin (Sigma‐Aldrich) for 1 week. The knockdown efficiency was confirmed by qRT‐PCR and Western Blot. The target sequences of selected shRNAs are listed in Table S2.

### 2.6 RNA isolation and qRT‐PCR

RNA was isolated by the NucleoSpin® RNA isolation kit of Macherey‐Nagel (Dueren, Germany) and reverse‐transcribed into cDNA using the PrimeScript™ RT Master Mix (Perfect Real Time, Takara, cat# RR036A), according to the manufacturer's instructions. mRNA quantification by qRT‐PCR was performed using SYBR™ Green PCR Master Mix (ThermoFisher) in a StepOnePlus™ Real‐Time PCR System (Applied Biosystems), using 12.5 ng cDNA per reaction, with the following conditions: 50°C for 2 minutes, 95°C for 2 minutes, then 38 cycles of 95°C for 15 seconds, 58°C for 15 seconds, 72°C for 1 minutes, followed by the Melt Curve stage of 95°C for 15 s, 60°C for 1 minute and a 0.7°C step‐wise increase until 95°C was reached. Means of technical replicates were used for calculation of gene expression by 2^−ΔΔT^ method. The geometric mean of three housekeeping genes (*HPRT1*, *GUSB*, *PMM1*) was used to normalize gene expression in patient samples. For experimental models, *GAPDH* was used as a reference gene to normalize target gene expression. Primer sequences used for qPCR of mRNA are included in Table S3. For tRNA quantification, we used the isolation protocol and primer sequences that we previously described.^6^

### 2.7 Western blotting

Cells were lysed in Laemmli sample buffer with 0.1 M DTT and heated for 5‐10 minutes at 95°C, followed by loading and separation on an 8%‐15% sodium dodecyl sulphate‐polyacrylamide gels. After 90 minutes running at 100 V, proteins were electrophoretically transferred onto a polyvinylidene difluoride membrane (Invitrogen) for 1.5 hours with an electric current of 250 mA. Subsequently, the membrane was blocked with blocking buffer (Li‐COR, Lincoln, USA) mixed with PBST in the ratio of 1:1. This was followed by overnight incubation with rabbit anti‐KARS (Sanbio BV, #14951‐1‐AP), anti‐Cleaved‐Caspase 3 (CST, #9664S), and mouse anti‐β‐actin (Santa Cruz, #SC‐47778) at 4°C. The membrane was washed three times with PBST, which was followed by incubation for 1 hour with anti‐rabbit (1:10 000) or anti‐mouse (1:5000) IRDye conjugated secondary antibodies (Li‐COR, Lincoln, USA) at room temperature. Blots were assayed for actin content as standardization of sample loading and scanned and quantified by Odyssey infrared imaging (Li‐COR, Lincoln, USA). The results were analysed with Image Studio software.

### 2.8 Analysis of cell cycle

Huh7 and SNU398 cells (5 × 10^5^/well) were plated in 6‐well plates and incubated overnight to attach to the bottom, and then serial concentrations of Lysine were added. After incubated 3 days, cells were harvested and fixed in cold 70% ethanol for 2 hours at 4°C. The cells were washed twice with PBS and incubated with 50 µL RNase (100 µg/mL) at 37°C for 30 minutes. Finally, cells were incubated with 250 µL Propidium Iodide (PI, 50 µg/mL) at room temperature for 5 minutes. The samples were analysed immediately by flow cytometry using a FACS Canto (BD Biosciences), and the cell cycle was analysed by using FlowJo_V10 software.

### 2.9 Colony and organoid formation assay

For colony formation assay, HCC cells were harvested and suspended in medium, then seeded into 6‐well plates (1000 cells/well) and the medium was refreshed every 4 days. After 2 weeks of culture, formed colonies were washed with PBS and fixed by 70% ethanol, followed by counterstaining with crystal violet (Sigma‐Aldrich) and washed with PBS. Colony sizes were measured microscopically by digital image analysis.

For the organoid formation assay, intact organoids were first digested into single cells, and then seeded into 48‐well plates (3000‐5000 cells/well). After 2 weeks, the sizes and numbers of formed organoids were calculated.

### 2.10 MTT and Alamar Blue assays

HCC cells were seeded in 96‐well plates at a concentration of 3 × 10^3^ cells/well in 100 µL medium. Upon indicated treatment, cell viability was analysed by adding 5 mg/mL MTT for 4 hours. After discarding the cell supernatant, 100 µL DMSO was added followed by 10 minutes shaking. Absorbance was determined using the microplate absorbance reader (Bio‐Rad, Hercules, CA, USA) at a wavelength of 490 nm.

Organoids were split in the ratio of 1:10 for daily culture and seeded in 24‐well plates. On the third or seventh day of indicated treatment, organoids were incubated with Alamar Blue (Invitrogen, 1:20 in Advanced/F12 DMEM) for 4 hours, and the medium was collected for measurement of the metabolic activity of the organoids. Absorbance was determined by a fluorescence plate reader (CytoFluor® Series 4000; Perseptive Biosystems) at the excitation of 530/25 nm and emission of 590/35 nm.

### 2.11 Cell migration assay

Cells (5 × 10^5^/well) were collected and suspended in 300 µL of serum‐free medium after the indicated treatment for 48 hours. Cells were placed into the upper chamber of a 24‐transwell (8‐µm pore size; Corning, #353097), and 700 µL of DMEM containing 20% FBS was added into the lower chamber. Cells were allowed to migrate to the bottom chamber for 24 hours. Next, cells that remained on the apical side of the chamber were gently scraped off using wetted cotton swabs. Subsequently, cells were fixed with 4% paraformaldehyde solution for 30 minutes and stained with 0.1% crystal violet for 30‐60 minutes. Finally, the cells were observed with a microscope, and images were obtained.

### 2.12 Statistics

All statistical analyses were performed using Graphpad (Version 5 for Windows, San Diego, CA) and R Statistical software (Version 3.6.1 for Windows, Foundation for Statistical Computing, Vienna, Austria). The expression level of genes between HCC and TFL patient tissues were compared using the Wilcoxon matched pairs test. For functional experiments, potential differences between the experimental groups were analysed using the Mann‐Whitney test. The correlation analysis was performed in RStudio with the ‘corplot’ package, using Spearman's correlation coefficient. For creating heatmaps, RStudio was used with the ‘gplots’ and ‘pheatmap’ packages. Survival analysis was performed by the Kaplan‐Meier method and the Cox proportional hazards model. Time to the event, either HCC‐specific death or HCC‐recurrence, was calculated from the day of surgery. If the event of interest did not occur, data were censored at the time of the last follow‐up, or if a patient underwent liver transplantation, at the time of liver transplantation. Used statistical tests are indicated in the figures. *P*‐values < 0.05 were considered significant, and were indicated with a single asterisk. *P*‐values < 0.01 were indicated with double asterisks and *P*‐values < 0.001 were indicated with triple asterisks.

## 3 RESULTS

### 3.1 Profiling the tRNAome landscape in patient HCC tumours identified the potential clinical significance of tRNA‐Lys‐CUU

**Figure 1:**
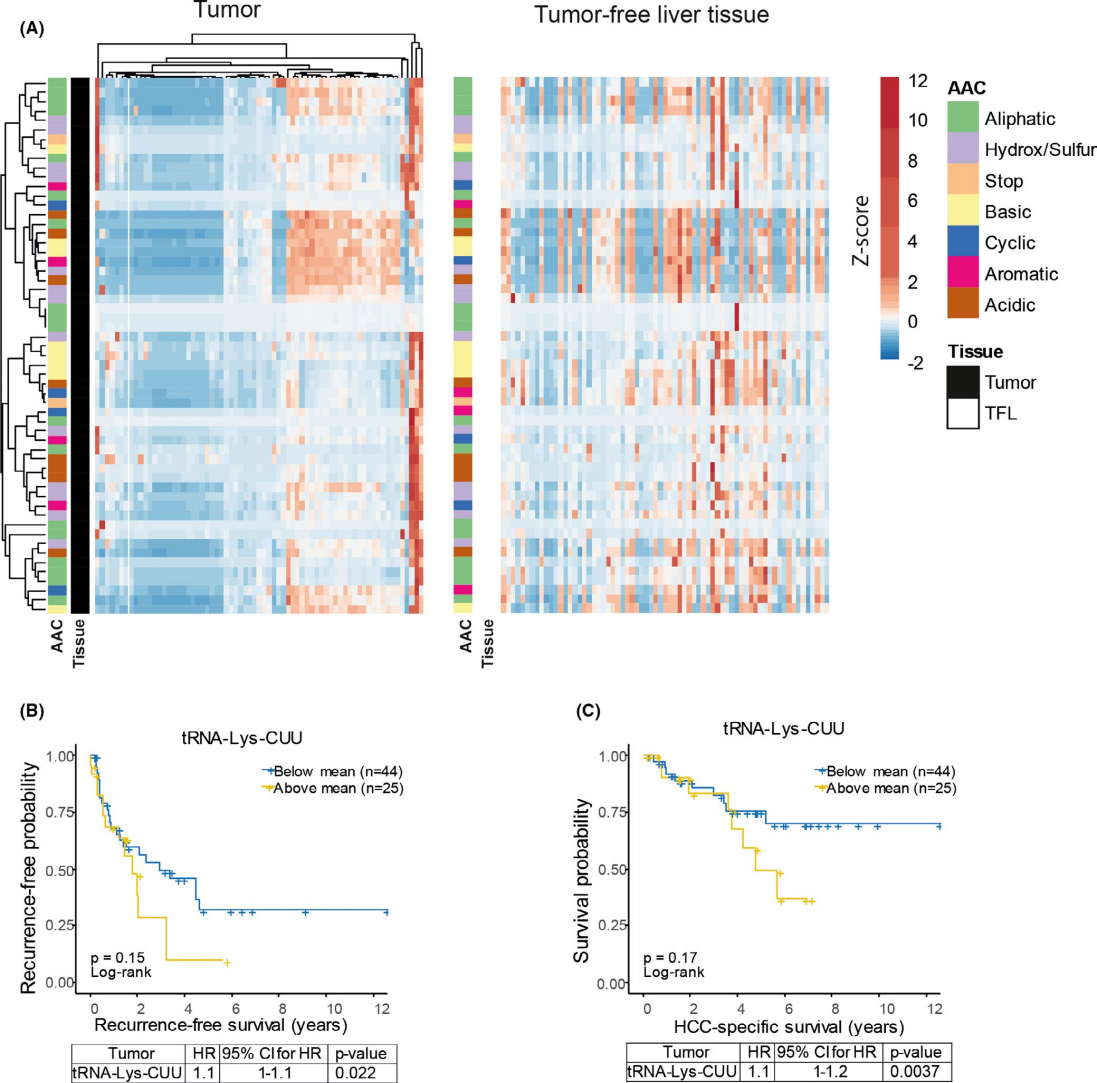
Profiling tRNAome in HCC patients identified tRNA‐Lys‐CUU having potential clinical significance. (A) Heatmap showing the mean expression (z‐score normalized per tRNA species, represented in colour, as indicated in the legend) of 57 tRNA in tumour and TFL tissues respectively. Both rows (tRNA) and columns (patient samples) of tRNAs expressed in tumours (left panel) are hierarchically clustered using correlation distance and complete linkage. Rows and columns of tRNAs expressed in TFL (right panel) were ordered according to the rows and columns in the tumour heatmap. (B and C) Kaplan‐Meier analysis and Cox regression analysis of tumour recurrence (B) and HCC‐specific survival (C) in relation to tRNA‐Lys‐CUU expression. The panels below the survival graphs show the results of univariate Cox regression analysis. HR, Hazard Ratio; AAC, amino acid class; HCC, hepatocellular carcinoma; TFL, tumour free liver; CI, confidence interval

Mature tRNAs with a CCA tail are charged with amino acids to elongate protein synthesis and are thus biologically most relevant. To focus on this functional species of tRNAs, we recently developed a specific qRT‐PCR quantification assay involving the removal of attached amino acids and a universal adaptor ligated to the CCA tail.^6^ We profiled the tRNAome consisting of 57 mature tRNA species in 69 pairs of tumours and matched TFL tissues of HCC patients. The heatmap depicted in Figure 1A shows the relative expression levels of 57 mature tRNAs in 69 pairs of tumour and TFL tissues. Significantly differentially expressed tRNAs between tumour and TFL tissues were listed in Table S4, showing that many tRNAs were down‐regulated in tumours. Interestingly, the patterns of tRNA expression were closely clustered and correlated based on the individual tissues (Figure S2A), suggesting that different members of the tRNAome function cooperatively in protein translation. Next, the expression of each individual tRNA in tumour tissues was further analysed to identify potential associations with clinical features. We found that tRNA‐Lys‐CUU has potential clinical relevance. Kaplan‐Meier analysis showed that high expression of tRNA‐Lys‐CUU in tumour appears to associate with more tumour recurrence (Figure 1B) and worse patient survival late after tumour resection (Figure 1C), although not statistically significant. Interestingly, univariate Cox regression analysis indicated that high levels of tRNA‐Lys‐CUU in tumour were significantly associated with increased rates of tumour recurrence (HR 1.1; *P* = .022) and HCC‐specific death (HR 1.1; *P* = .0037) (Figure 1B,C; lower panels). In multivariate analysis together with clinicopathological characteristics, tRNA‐Lys‐CUU expression in tumour was an independent negative prognostic factor for both HCC‐specific survival and HCC recurrence (Figure S2B). As TFL tissues from HCC patients often show various signs of liver pathology, we also included healthy controls of liver tissues obtained from organ donor livers. The expression of tRNA‐Lys‐CUU was significantly higher in HCC tumour tissues compared with the healthy liver tissues (Figure S2C).

### 3.2 Elevated expression of Lysyl‐tRNA synthetase in patient HCC tissues

**Figure 2:**
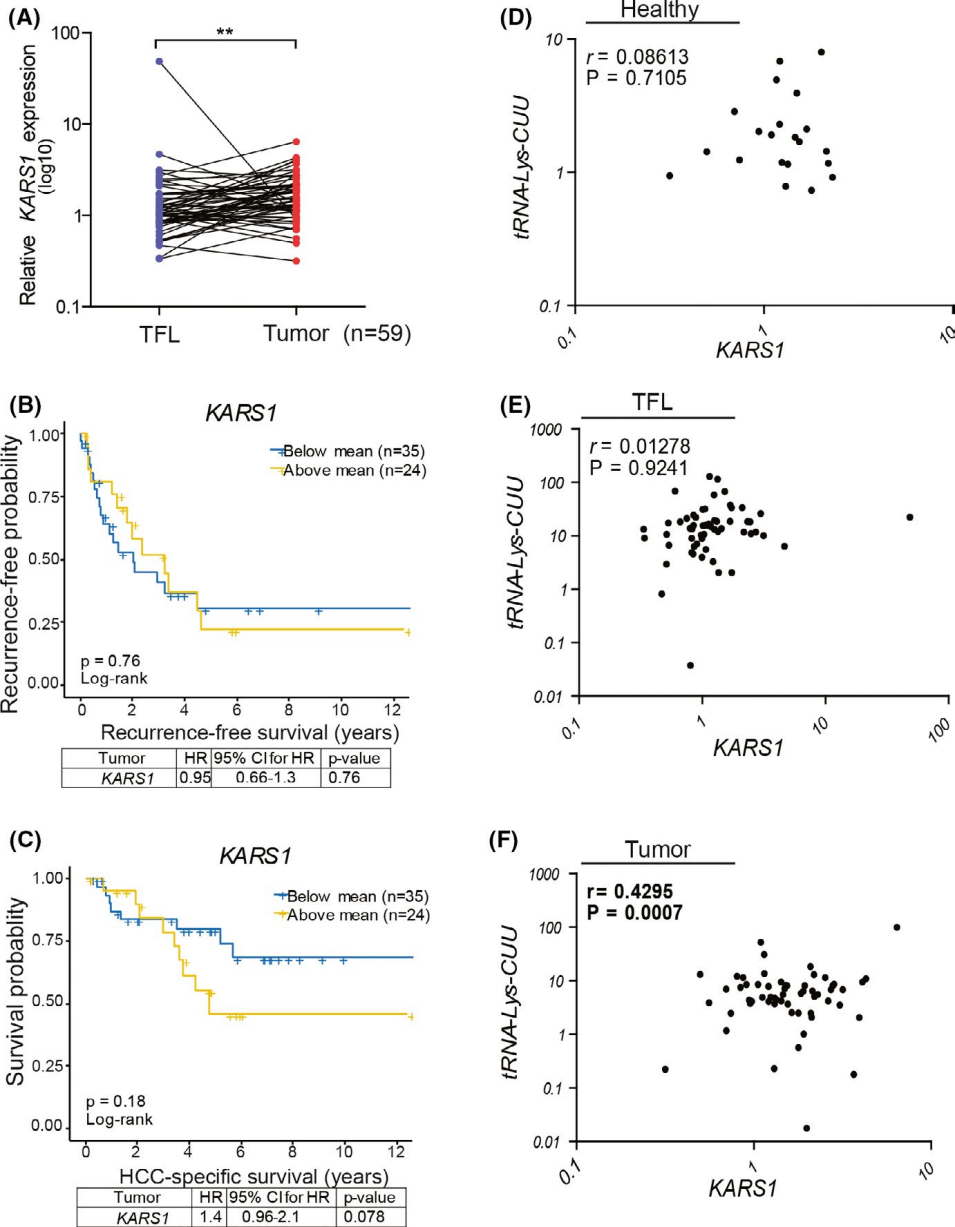
*KARS1* expression and clinical relevance in HCC patients. (A) mRNA expression of *KARS1* in HCC tumours compared to paired TFL tissues (n = 59). (B and C) Kaplan‐Meier analysis of tumour recurrence (B) and HCC‐specific survival (C) in relation to *KARS1* expression in tumours. The panels below the survival graphs show the results of univariate Cox regression analysis. (D‐F) Correlation analysis between the expression levels of tRNA‐Lys‐CUU and *KARS1* in healthy organ donor liver tissues (D, n = 21), TFL (E) and paired HCC tissues (F, n = 59) respectively. **P < .01, by the Wilcoxon matched pairs test. *KARS1*, Lysyl‐tRNA Synthetase; HCC, hepatocellular carcinoma; TFL, tumour free liver; HR, Hazard Ratio; CI, confidence interval

Lysyl‐tRNA synthetase (KARS), encoded by *KARS1*, catalyses the charging of lysine to tRNA‐Lys‐CUU and tRNA‐Lys‐UUU.^16^ We found that mRNA expression levels of *KARS1* were significantly higher in HCC tumour tissues compared with paired TFL tissues (n = 59) (Figure 2A; Figure S3A), and this was further confirmed by analysis of the publically available GEPIA database (Figure S3B). When analysing the association with clinical outcomes, we found no evidence for a relation with tumour recurrence (Figure 2B). However, there was a trend of association with patient survival. In univariate Cox regression analysis patients with high *KARS1* expression seemed to have an increased death rate (HR 1.4; Figure 2C), although statistically not significant (*P* = .078). Importantly, multivariate analysis revealed a significantly increased risk of death for high KARS1 expressers (HR 1.66; *P* = .021) (Figure S3C). Altogether, these associations between the expression levels of tRNA‐Lys‐CUU and *KARS1* in tumour tissues and prognosis of HCC patients indicate a potential clinical relevance for the interface of charging lysine with its tRNA in liver cancer. In addition, we found a significant positive correlation of tRNA‐Lys‐CUU and *KARS1* in tumour tissues (Figure 2F), but not in healthy liver tissues or TFL tissues (Figure 2D,E). This promoted us to further explore their functions in experimental models.

### 3.3 Knockdown of KARS inhibits HCC cell growth

**Figure 3:**
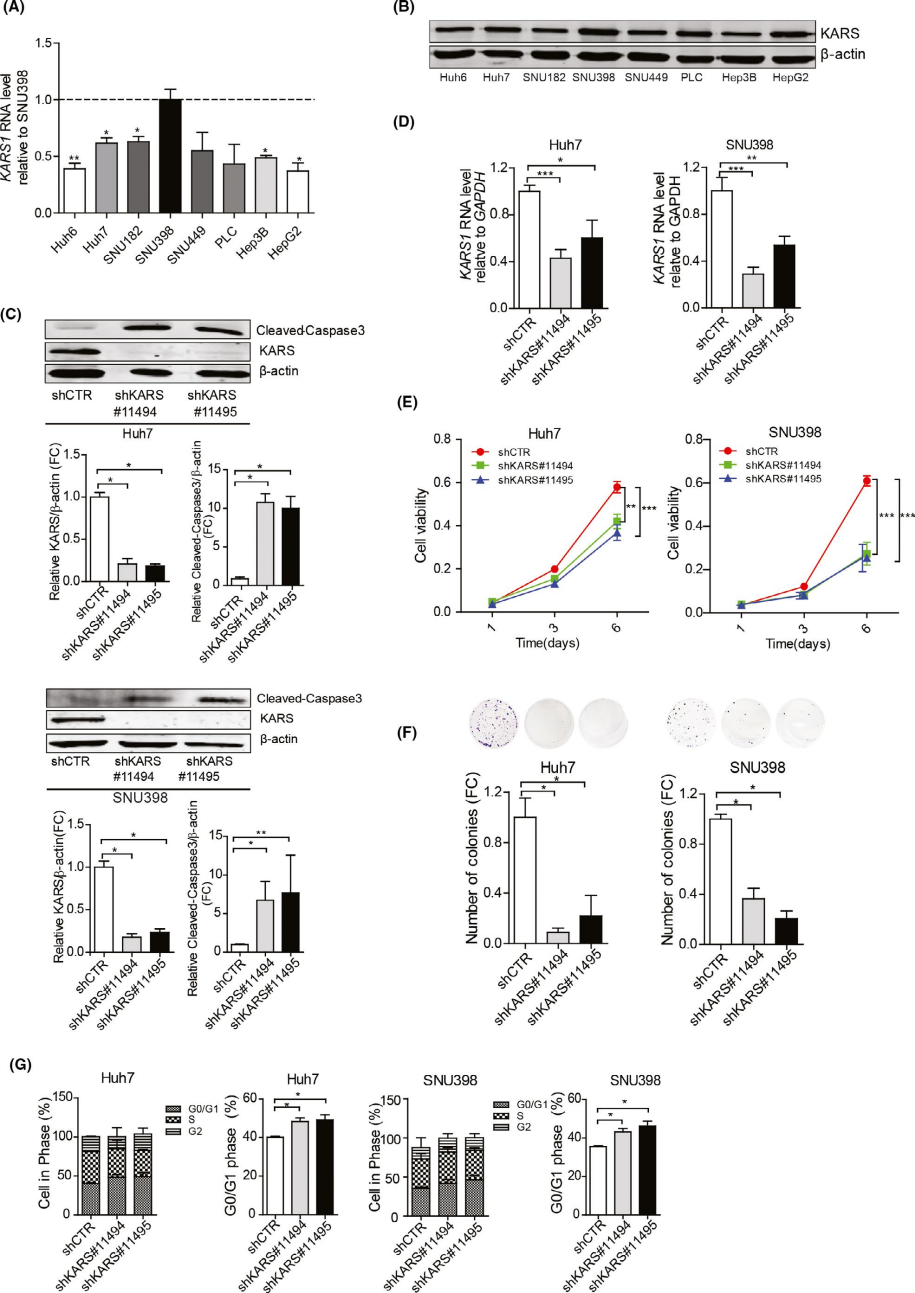
The effects of KARS knockdown on HCC cell lines. (A) Relative expression of *KARS1* mRNA in HCC cell lines compared to SNU398 (n = 4‐6). (B) KARS protein expression in HCC cell lines. (C) Protein expression levels of KARS and cleaved‐Caspase 3 in Huh7 and SNU398 HCC cells following shRNA mediated knockdown of KARS. The intensity was quantified relative to β‐actin (n = 4‐8). (D) *KARS1* mRNA expression following shRNA mediated knockdown quantified by qRT‐PCR (n = 4‐10). (E) Effects of KARS knockdown on cell growth measured by MTT assay following 1, 3 and 6 days of culture (n = 11, 22). (F) KARS knockdown affects the number of single cell formed colonies as assayed 2 weeks following seeding (n = 5). (G) The effect of KARS knockdown on cell cycling was measured by Propidium Iodide staining, and the fraction of cells in G0/G1 was quantified (n = 4‐6). Quantification of C, D and F data of knockdown groups were relative to the shCTR group. Data are presented as mean ± SEM. *P < .05; **P < .01; ***P < .001, by the Mann‐Whitney test. HLO, healthy liver organoids; HCC, hepatocellular carcinoma; FC, fold change; *KARS1*, Lysyl‐tRNA Synthetase

To study the functionality of KARS in liver cancer cells, we first examined *KARS1* expression at mRNA and protein levels (Figure 3A,B), and tRNA‐Lys‐CUU expression across eight HCC cell lines (Figure S4A). All HCC cell lines expressed higher *KARS1* RNA levels, and all except one expressed higher levels of tRNA‐Lys‐CUU than healthy liver organoids. Huh7 and SNU398 cell lines with high levels of *KARS1* and tRNA‐Lys‐CUU expression were selected for further experimentation. We used the lentiviral RNA interference approach to investigate the effects of silencing KARS on HCC cells, and selected the two shRNAs with optimal efficiency for KARS knockdown (Figure S4B,C; Figure 3C,D). KARS knockdown attenuated the overall growth of Huh7 and SNU398 cells (Figure 3E), and dramatically inhibited single cell‐based colony formation (Figure 3F). Silencing of KARS arrested cell cycling at the G0/G1 phase (Figure 3G) and induced cell apoptosis as indicated by drastically increased levels of cleaved‐Caspase 3 protein (Figure 3C).

### 3.4 Lysine deprivation inhibits HCC cell growth and migration

**Figure 4:**
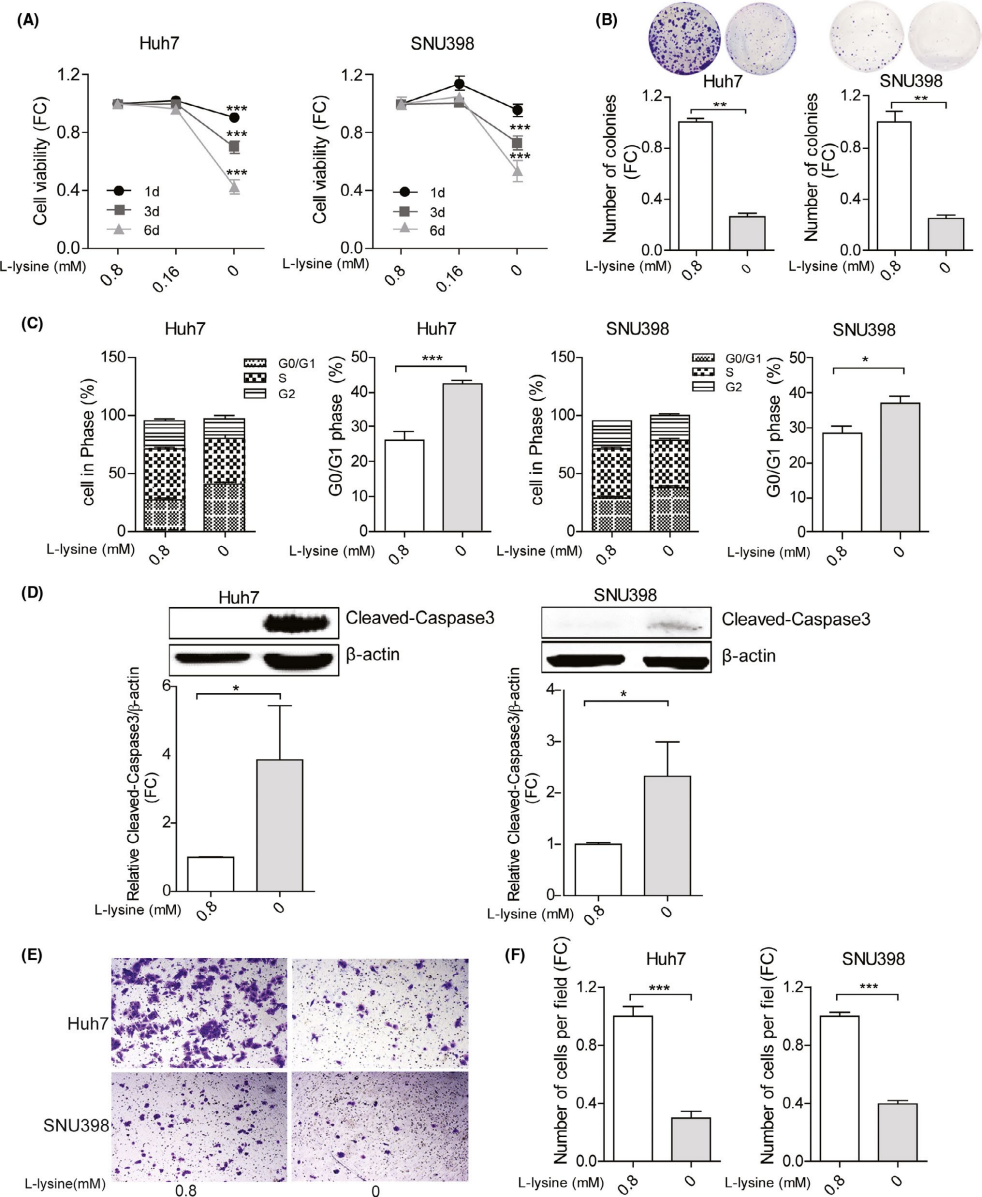
The effects of lysine on HCC cell lines. (A) The effects of lysine on cell growth measured by MTT assay following 1, 3 and 6 days of culture (n = 20). (B) Lysine affects the number of single cell‐derived colonies as assayed 2 weeks following seeding (n = 6). (C) The effects of lysine on cell cycling was measured by Propidium Iodide staining, and the fraction of cells in G0/G1 was quantified (n = 6‐9). (D) Protein expression levels of cleaved‐Caspase 3 in Huh7 and SNU398 HCC cells. The intensity was quantified relative to β‐actin (n = 5). Huh7 and SNU398 cells were cultured with or without lysine for 3 days. (E) Representative images of migrating cells with or without lysine and (F) quantification of the number of migrating cells (n = 10‐20). Quantification of A, B, D and F data without lysine groups were relative to with lysine group. Data are presented as mean ± SEM. *P < .05; **P < .01; ***P < .001, by the Mann‐Whitney test. HCC, hepatocellular carcinoma; FC, fold change

Lysine is the substrate which is ligated by KARS to tRNA‐Lys‐CUU. We first examined the effects of complete deprivation of each of the nine essential amino acids in SNU398 cells. We found that deprivation of any of the nine amino acids had some effects on cell growth, but lysine deprivation exerted the strongest inhibitory effect (Figure S5A). In single cell‐based colony formation assay, deprivation of lysine, methionine or valine but not any of the other six amino acids had significant inhibition, and lysine deprivation again exerted the strongest effect (Figure S5B). These results collectively indicated lysine is most essentially required. Next, compared to the normal concentration (0.8 mM) of lysine in cell culture medium, lowering the concentration to 0.16 mM had minimal effects on HCC cell growth (Figure 4A). However, complete deprivation of lysine dramatically inhibited overall cell growth (Figure 4A) and single cell‐based colony formation (Figure 4B). This was associated with increased G0/G1 cell cycle arrest (Figure 4C), and increased cell apoptosis indicated by the dramatic increase of Caspase 3 cleavage (Figure 4D). Interestingly, lysine deprivation also attenuated HCC cell migration in the transwell system, an assay recapitulating some features of cancer cell invasion and metastasis (Figure 4E,F).

### 3.5 Cladosporin, a pharmacological inhibitor of KARS, inhibits HCC cell growth and migration

**Figure 5:**
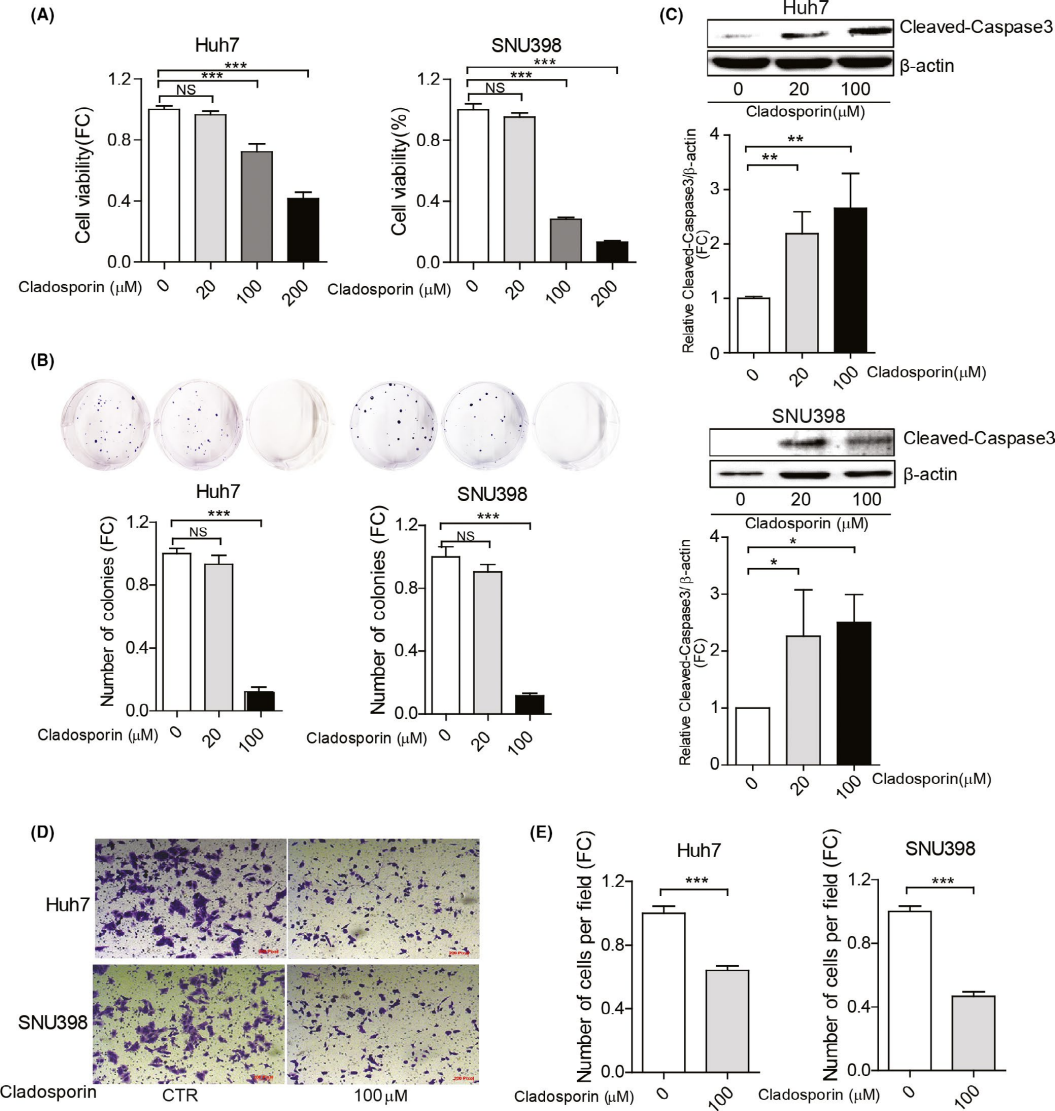
The effects of cladosporin treatment on HCC cell lines. (A) Huh7 and SNU398 cells were treated with different concentrations of cladosporin for 3 days. Cell viability was measured by MTT assay (n = 12). (B) Cladosporin affects the number of single cell‐derived colonies as assayed 2 weeks following seeding (n = 6‐8). (C) Protein expression levels of cleaved‐Caspase 3 in Huh7 and SNU398 cells, and the intensity was quantified relative to β‐actin (n = 5). Huh7 and SNU398 were treated with cladosporin for 3 days. (D) Representative images of migrating cells with cladosporin and (E) quantification of the number of migrating cells (n = 20‐25). Quantification of all data were relative to the negative control group. Data are presented as mean ± SEM. *P < .05; **P < .01; ***P < .001, by the Mann‐Whitney test. HCC, hepatocellular carcinoma; FC, fold change

As the biological process of charging lysine with tRNA sustains HCC cells, we next examined whether this can be therapeutically targeted. We evaluated the effects of the well‐established KARS pharmacological inhibitor cladosporin, an antifungal antibiotic isolated from *Cladosporium cladosporioides* and *Aspergillus flavus*. Recently we accomplished a scalable synthesis of the active natural product cladosporin, producing more than 2 g of the compound.^17^ We found the 50% inhibitory concentrations of cladosporin to be 104 µM and 70 µM in Huh7 and SNU398 cells respectively (Figure S6). Treatment with different concentrations of cladosporin (0‐200 µM) dose‐dependently inhibited Huh7 and SNU398 cell growth (Figure 5A). Consistently, cladosporin significantly inhibited single cell‐based colony formation (Figure 5B), induced Caspase 3 cleavage (Figure 5C) and affected cell migration (Figure 5D,E). Combination of cladosporin treatment and *KARS1* gene silencing further augmented the inhibitory effects (Figure S7A,B).

### 3.6 Clinical relevance of KARS expression in CC patients and inhibition of patient CC organoids growth by lysine deprivation or treatment with cladosporin

**Figure 6:**
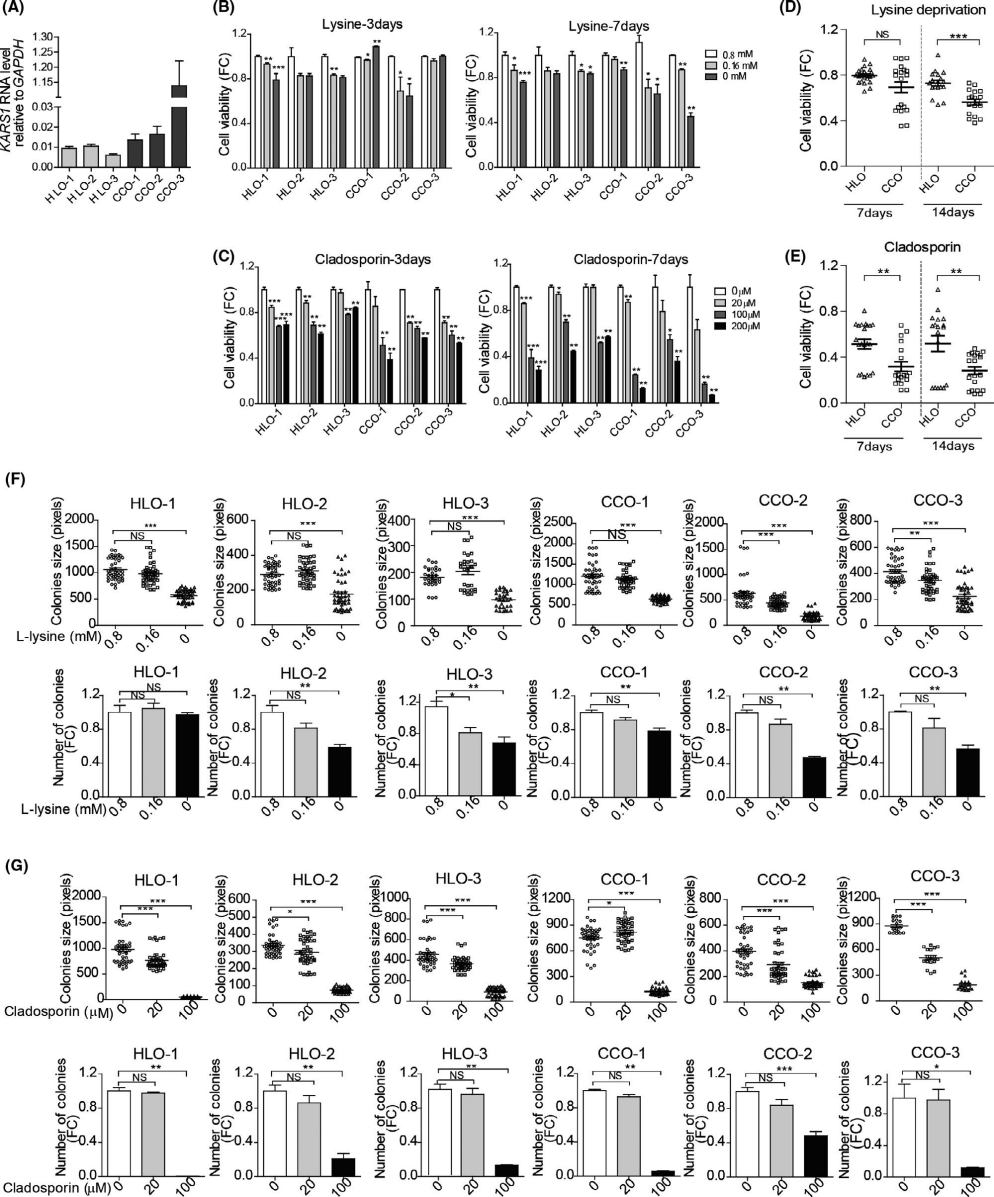
Lysine deprivation or treatment with cladosporin affects the growth of patient‐devied CC organoids. (A) Relative expression of *KARS1* mRNA in patient CC organoids and human healthy liver organoids (HLO) (n = 5‐11). Effects of (B) lysine deprivation or (C) cladosporin treatment on cell viability were measured by Alamar blue assay after culturing 3 or 7 days (n = 4‐12). Organoids were treated with (D) lysine deprivation or (E) cladosporin (100 µM) for 7 or 14 days. The results depicts the mean viability of CC organoids (CCO‐1, CCO‐2, CCO‐3) compared with the mean viability of healthy liver organoids (HLO‐1, HLO‐2, HLO‐3) (n = 18‐24). (F) Lysine deprivation or (G) cladosporin treatment affects organoid initiation as assayed 2 weeks following seeding. The number (n = 4‐10) and size (n = 18‐45) of organoids after culturing for 14 days were calculated. Quantification of the data target groups were relative to the negative control group. Data are presented as mean ± SEM and dots represent individual organoid cultures. *P < .05, **P < .01, ***P < .001, by the Mann‐Whitney test. CCO, cholangiocarcinoma organoid; HLO, human liver healthy organoid; FC, fold change

We have included three batches of healthy human liver organoids and three batches of CC organoids. Because organoids are very difficult to be cultured from HCC tissues, we used liver tumour organoids cultured from CC patients,^18^ of which the *KARS1* expression levels were shown in Figure 6A. Importantly, similar to what we found in HCC patients, *KARS1* expression was significantly higher in patient CC tumour tissues based on the GEPIA database. However, in this small CC cohort, *KARS1* expression was not significantly associated with patient's prognosis (Figure S8). Thus, we tested the effects of lysine deprivation or cladosporin on patient‐derived CC organoids in 3D culture. We found significant growth inhibition of three independent batches of CC organoids, with the effects being more robust after 7 days of treatment (Figure 6B,C; Figure S9A,B). In parallel, we examined the effects on three independent batches of healthy human liver organoids. Although lysine deprivation or cladosporin treatment also inhibited growth of the healthy liver organoids, the effects were significantly stronger on the CC organoids (Figure 6D,E; Figure S9C,D). Next, we tested the effects on organoid initiation. Lysine deprivation or cladosporin treatment significantly inhibited the number and the size of initiated CC organoids from single cells (Figure 6F,G; Figure S9E‐H). Interestingly, the responsiveness varies among organoids derived from different patients (Figure 6). Taken together, the biological process of charging tRNA with lysine sustains HCC cell growth and can be therapeutically targeted as demonstrated in cell culture and patient organoid models.

## 4 DISCUSSION

tRNAs perform central functions in protein synthesis. Intuitively, dysregulation of tRNA expression is expected to associate with pathogenesis, such as cancer development.^19^, ^20^, ^21^ However, because of the intrinsic complexity and lack of easy techniques for quantification, the role of tRNAs in cancer has rarely been studied. The recently launched tRNAscan‐SE program that can accurately identify genomic tRNA sequences has facilitated the development of tRNA‐based quantification methods.^22^ High‐throughput sequencing techniques have been used to quantify the tRNAome at transcriptional level,^23^ but they are highly dependent on programming and very specialized reagents. We have recently developed a novel qPCR method that specifically quantifies mature tRNAs.^6^ We think this form of tRNAs is functionally most relevant as these mature tRNAs are directly charged with amino acids and thus facilitate protein synthesis.

In this study, we first profiled the tRNAome consisting of 57 mature tRNA species in 69 pairs of tumours and matched TFL tissues of HCC patients. We surprisingly observed that many tRNAs are down‐regulated in HCC tumour compared to TFL. This is different from previous studies comparing tRNA expression between cancer cell lines and noncancerous cell lines or human cancer and healthy non‐cancer tissues.^21^ Of note, TFL tissues represent a unique intermediate state between healthy liver tissue and tumour tissue. In many HCC patients, TFL is chronically inflamed and cirrhotic (Table 1). Indeed, expression levels of tRNA‐Lys‐CUU in both tumours and tumour‐free liver tissues of HCC patients were significantly enhanced compared to healthy livers. Interestingly, we found that the expression patterns of tRNAs were closely clustered and correlated within individual tissues, suggesting that they function cooperatively and collectively in protein synthesis. Among these tRNAs, we found that high expression of tRNA‐Lys‐CUU in HCC tissue appears to associate with poor clinical outcomes, although the effects were moderate and our cohort is too small to draw firm conclusions.

Recently, amino acid deprivation therapy has been widely explored as potential anti‐cancer strategy. Among different approaches in interrupting amino acid metabolism in cancer cells, enzymatic depletion is the most promising strategy. This results in deprivation of these exogenously supplied nutrients, and thus inhibits tumour growth.^24^ Deprivation of amino acids including asparagine,^25^ arginine,^26^ glutamine,^27^ methionine^28^, ^29^ and phenylalanine^30^ has been tested for cancer treatment in experimental models. As we have observed that high expression levels of tRNA‐Lys‐CUU potentially associates with poor outcomes in HCC patients, we tested whether deprivation of the substrate lysine functionally affects HCC cells in experimental models. Lysine deprivation inhibited overall cell growth, migration and single cell‐based colony formation, and induced cell cycling arrest and cell apoptosis in two HCC cell line models. These effects were further confirmed in 3D cultured patient CC organoids. Importantly, we found that HCC cells are more sensitive to deprivation of lysine compared to other essential amino acids. Our findings together with those of previous studies^13^, ^25^ suggest the potential of developing anti‐cancer strategies based on amino acid deprivation. However, the dependency of particular amino acid varies among different cancer types, and normal cells also require these amino acids to survive. Therefore, which amino acid to be targeted and which therapeutic modality to be used should be studied further.^31^

The structures and functions of ARSs that catalyse the charging of amino acids to cognate tRNAs have been widely studied. A systematic analysis of the expression of ARSs has identified that many of them are highly expressed in tumours and form networks with cancer‐associated genes. This also supports the fact that protein synthesis is universally accelerated in proliferating malignant cells.^32^, ^33^ We found that the expression of *KARS1* was significantly upregulated in HCC compared to TFL tissues. Similar results were also observed in CC patients. We postulate that the abundance of KARS will enhance the efficiency of catalysing lysine to charge the tRNAs including tRNA‐Lys‐CUU. This will accelerate protein translation of genes enriched with cognate codons, therefore promoting cancer cell growth. This hypothesis is supported by our findings in two HCC cell lines that knockdown of KARS inhibited cell growth, migration and colony formation, and induced cell cycle arrest and apoptosis.

ARSs targeted drug development has mainly been examined in the field of infectious diseases by exploiting species‐specific structural diversity and catalytic activity.^34^ For example, ARS‐inhibitors mupirocin and AN2690 are FDA‐approved for treatment of gram‐positive bacterial skin infections and fungal nail infections respectively.^12^ With respect to oncology, ARSs represent overlooked targets for therapeutic development against cancer.^33^ In this study, we tested a specific pharmacological KARS inhibitor cladosporin, an antifungal antibiotic isolated from *Cladosporium cladosporioides* and *Aspergillus flavus*.^35^, ^36^ We found that cladosporin can effectively inhibit the growth of HCC cell lines and CC organoids, consistent with the results from KARS knockdown and lysine deprivation. Interestingly, the responsiveness to lysine deprivation or cladosporin varies among CC organoids derived from different patients. However, the activity of cladosporin towards human KARS is 100‐fold lower than towards KARS of the malaria parasite.^37^, ^38^, ^39^ Cladosporin is currently explored as a potential treatment for malaria, but whether it is also applicable for treating cancer remains uncertain.^17^ Another important consideration is that ARSs are essentially required for maintaining the physiological functions of normal cells. Targeting human ARSs for treating cancer is expected to cause substantial side effects. Indeed, in healthy human liver organoids, we observed that both lysine deprivation and cladosporin treatment resulted in notable inhibitory effects, although to a lesser extent than those in CC organoids. Based on our HCC cell lines and CC organoids, cells expressing higher level of KARS appear to be more sensitive to KARS or lysine targeted inhibition. Collectively, our results support the notion that ARSs are viable targets for anti‐cancer drug development, but this is just the start in unveiling their biological functions in cancer and exploring possible therapeutic modalities.

In summary, we have profiled the tRNAome landscape in human HCC tumours and identified that high expression of tRNA‐Lys‐CUU in tumour is potentially associated with poor clinical outcomes. KARS expression is upregulated in both patient HCC and CC tumour tissues. In experimental models of HCC cell lines and CC patient‐derived organoids, biological or pharmacological targeting of the interface of charging lysine to tRNA‐Lys‐CUU inhibits cancer cell growth and migration. These findings bear important implications of exploring such unexplored territories for developing novel therapeutics against liver cancer.

## CONFLICTS OF INTEREST

The authors declare no competing interests.

## AUTHOR CONTRIBUTIONS

RZ, QP, MPP and XO designed the research. RZ, XO, and BM designed the mature tRNA assay. LN and JK provided patient samples and analysed data. PD and DSR synthesized natural product cladosporin and discussed the project. SS, JL, LW, PL, MMAV and LJWL contributed to the acquisition of human liver tissue samples for organoids culture. R.Z and YL contributed to the study of design and analysis data. QP, RS and MPP contributed to the study concept and discussed the data. QP and RZ drafted and revised the manuscript. LN, RS and JK extensively discussed the project and critically revised the manuscript. QP and XO share the co‐correspondence authorship.

## Supporting information

Supplementary Material
