# Supplementary material for: The biological process of lysine‐tRNA charging is therapeutically targetable in liver cancer
Source: Liver Int. 2020 Oct 20;41(1):206–19. doi: 10.1111/liv.14692 (PMC7820958; doi:10.1111/liv.14692)
Supplement: Supplementary file 1 — Supplementary Material [file LIV-41-206-s001.pdf]

Supplementary Figures and Tables

The biological process of lysine-tRNA charging is therapeutically targetable in liver cancer

Ruyi Zhang[1], Lisanne Noordam[1], Xumin Ou[1,2*], Buyun Ma[1], Yunlong Li[1], Pronay Das[3], Shaojun Shi[4], Jiaye Liu[1], Ling Wang[1], Pengfei Li[1], Monique M. A. Verstegen[4], D. Srinivasa Reddy[3], Luc J. W. van der Laan[4], Maikel P. Peppelenbosch[1], Jaap Kwekkeboom[1], Ron Smits[1], Qiuwei Pan[1*]

1. Department of Gastroenterology and Hepatology, Erasmus MC-University Medical Center, Rotterdam, The Netherlands.
2. Institute of Preventive Veterinary Medicine, Sichuan Agricultural University, Chengdu, China.
3. Organic Chemistry Division, CSIR-National Chemical Laboratory, Pune, India.
4. Department of Surgery, Erasmus MC-University Medical Center, Rotterdam, The Netherlands.

* These authors contributed equally to this paper

^{1}H NMR of Cladosporin in 400 MHz

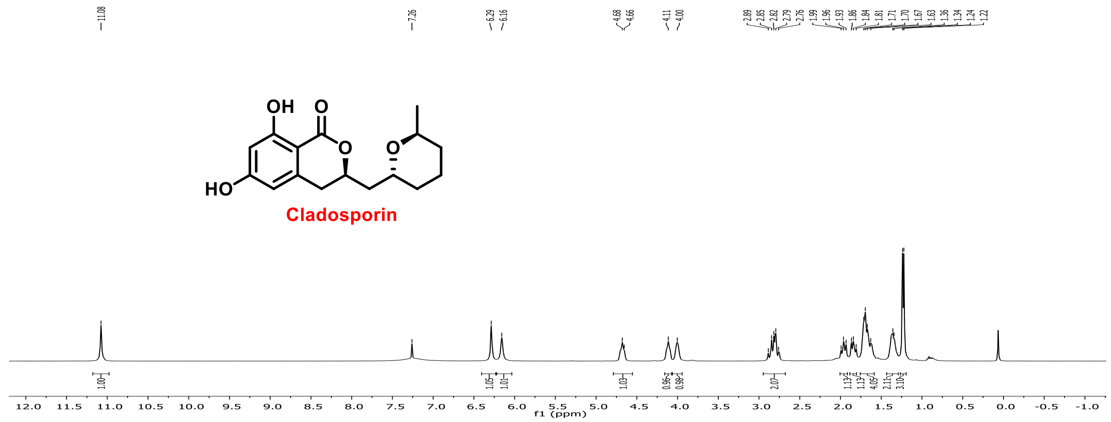

^{1}H NMR (400 MHz, $CDCl_3$) δ 11.08 (s, 1H), 6.29 (s, 1H), 6.16 (s, 1H), 4.68-4.66 (m, 1H), 4.11 (s, 1H), 4.00 (s, 1H), 2.89 – 2.76 (m, 2H), 1.90 (dt, J = 23.0, 12.8 Hz, 2H), 1.68 (dd, J = 21.9, 12.2 Hz, 4H), 1.36 -1.34 (m, 2H), 1.23 (d, J = 6.4 Hz, 3H).

^{13}C NMR of Cladosporin in 100 MHz

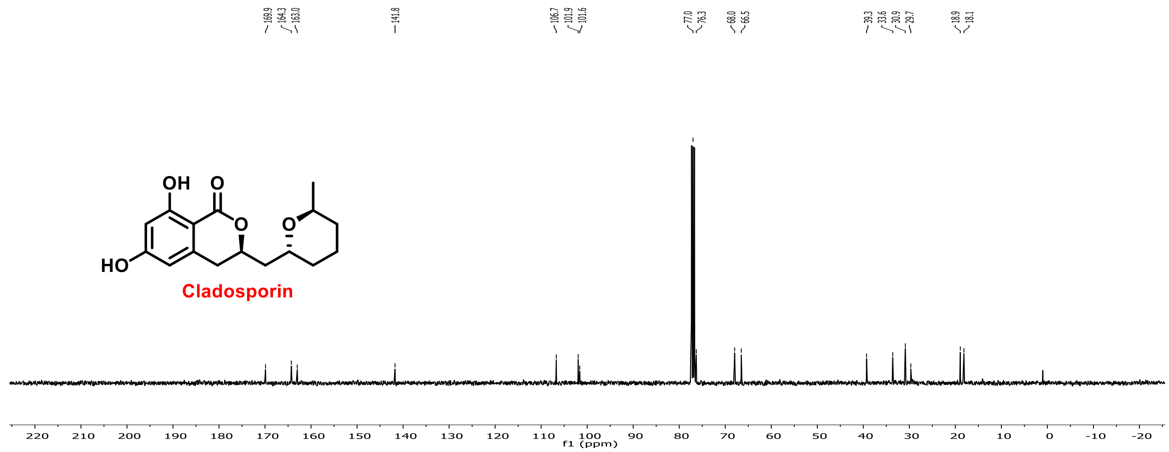

^{13}C NMR (100 MHz, $CDCl_3$) δ 169.9, 164.3, 163.0, 141.8, 106.7, 102.0, 101.6, 76.3, 68.0, 66.5, 39.3, 33.6, 30.9, 29.7, 18.9, 18.1

Supplementary Figure 1 NMR for Cladosporin

A

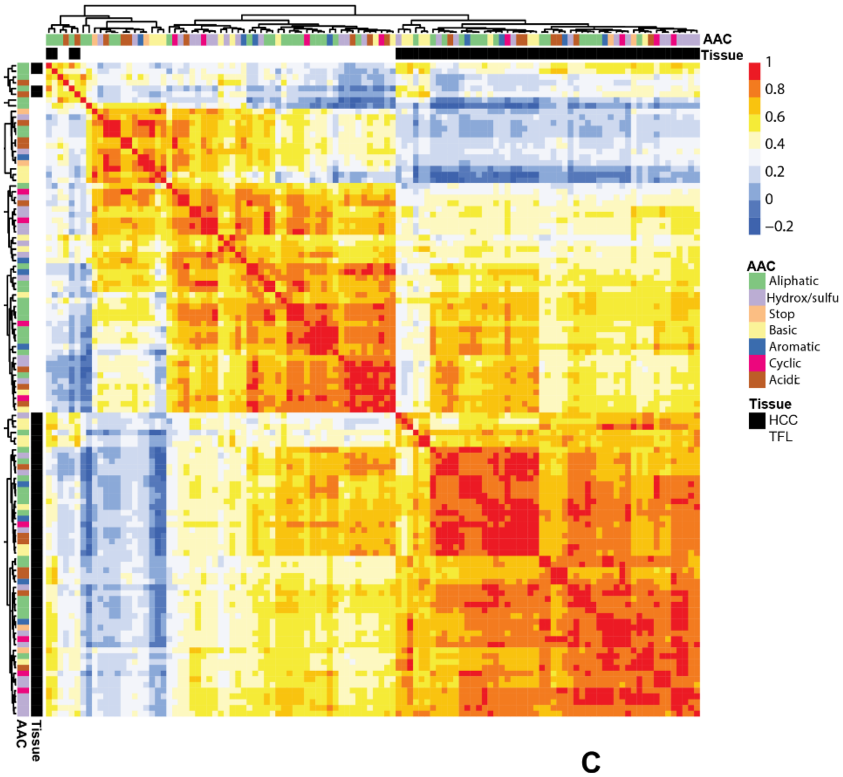

B

Survival rate, based on tRNA-Lys-CUU expression in HCC

	Univariate			Multivariate		
	HR	95% CI	p-value	HR	95% CI	p-value
Tumor *tRNA-Lys-CUU*	1.1	(1-1.2)	**0.0037**	1.14	1.048-1.235	**0.002**
AFP over 400 μg/L	1.9	(0.67-5.4)	0.23			
AFP over 1000 μg/L	4.1	(1.4-12)	**0.0087**	4.47	1.548-12.922	**0.006**
Patients with more than 1 tumor	0.83	(0.32-2.2)	0.71			
Patients with more than 2 tumors	1.8	(0.52-6.5)	0.34			
Patients with tumor over 5 cm	2	(0.7-5.7)	0.2			
Chronic viral hepatitis	2.4	(0.94-6.3)	0.066			
Vascular invasion	0.89	(0.34-2.3)	0.82			

Recurrence rata, based on tRNA-Lys-CUU expression in HCC

	Univariate			Multivariate		
	HR	95% CI	p-value	HR	95% CI	p-value
Tumor *tRNA-Lys-CUU*	1.1	1-1.1	**0.022**	1.09	1.026-1.16	**0.005**
AFP over 400 μg/L	1.7	(0.76-3.7)	0.2			
AFP over 1000 μg/L	2.4	(0.97-5.8)	0.059			
Patients with more than 1 tumor	1.5	(0.78-3)	0.21			
Patients with more than 2 tumors	4.3	(2-9.2)	**0.00019**	3.75	1.54-9.12	**0.004**
Patients with tumor over 5 cm	1.3	(0.66-2.6)	0.44			
Chronic viral hepatitis	2.6	(1.3-5)	**0.0047**	1.76	0.82-3.78	0.145
Vascular invasion	1.1	(0.55-2.3)	0.73			

C

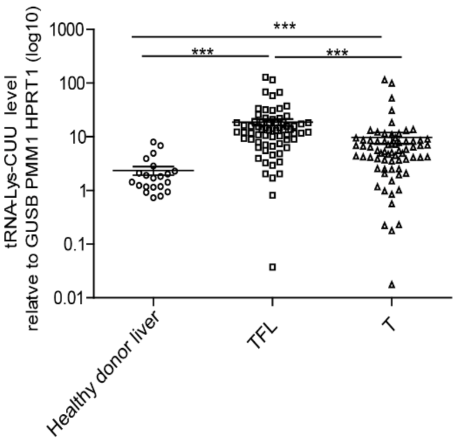

Supplementary Figure 2 (A) Heatmap showing the correlation patterns of 57 tRNA species in 69 pairs of human HCC and TFL tissues. (B) The expression of tRNA-Lys-CUU in organ donor liver tissues (n = 21), TFL and paired HCC tissues (n = 69), respectively. (C) Univariate and multivariate analysis of associations between clinicopathological risk factors and tRNA-Lys-CUU expression in tumor tissue and HCC-specific patient survival rate and cancer recurrence rate (n=69) in our HCC patient cohort. ***P <0.001, by the Mann-Whitney test. TFL: tumor free liver; T:HCC tumor.

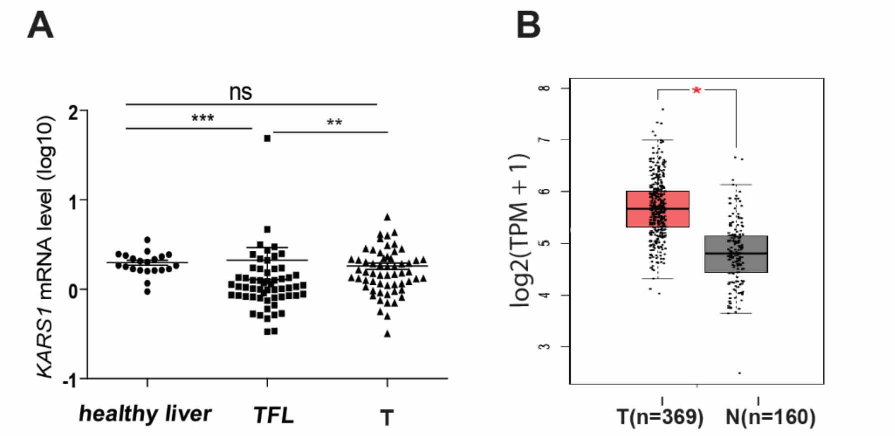

C

Survival rate, based on *KARS1* expression in HCC

	Univariate			Multivariate		
	HR	95% CI	p-value	HR	95% CI	p-value
Tumor *KARS1*	1.4	0.96-2.1	**0.078**	**1.66**	1.08-2.55	**0.021**
AFP over 400 μg/L	1.9	(0.67-5.4)	0.23			
AFP over 1000 μg/L	4.1	1.4-12	**0.008**	5.77	1.86-17.94	**0.002**
Patients with more than 1 tumor	0.78	(0.3-2.1)	0.62			
Patients with more than 2 tumors	1.8	(0.5-6.3)	0.38			
Patients with tumor over 5 cm	2.1	(0.74-6)	0.16			
Chronic viral hepatitis	2.4	(0.92-6.2)	0.073			
Vascular invasion	1.2	(0.48-3.2)	0.66			

Recurrence rata, based on *KARS1* expression in HCC

	Univariate		
	HR	95% CI	p-value
Tumor *KARS1*	0.95	(0.66-1.3)	0.76
AFP over 400 μg/L	1.6	(0.7-3.8)	0.26
AFP over 1000 μg/L	2.3	(0.88-6.1)	0.09
Patients with more than 1 tumor	1.4	(0.68-2.8)	0.38
Patients with more than 2 tumors	3.6	(1.6-8.3)	0.0025
Patients with tumor over 5 cm	1.4	(0.68-2.9)	0.37
Chronic viral hepatitis	2.5	(1.2-5)	0.011
Vascular invasion	0.97	(0.48-2)	0.94

Supplementary Figure 3 The mRNA expression levels of KARS1, and their relation with clinical outcomes.

(A) The mRNA expression levels of *KARS1* in organ donor liver tissues (n = 21), TFL and paired HCC tissues(n = 59).(B)The mRNA expression data of *KARS1* from GEPIA database, and red boxplot showed HCC tumor samples (n = 369), black boxplot showed Normal healthy liver samples (n = 160). (C) Univariate and multivariate analysis of associations between clinicopathological risk factors and *KARS1* expression in tumor tissue and HCC-specific patient survival rate and cancer recurrence rate (n=59) in our HCC patient cohort. *P<0.05; **P<0.01; ***P<0.001, by the Mann-Whitney test. TFL: tumor free liver; T:HCC tumor.

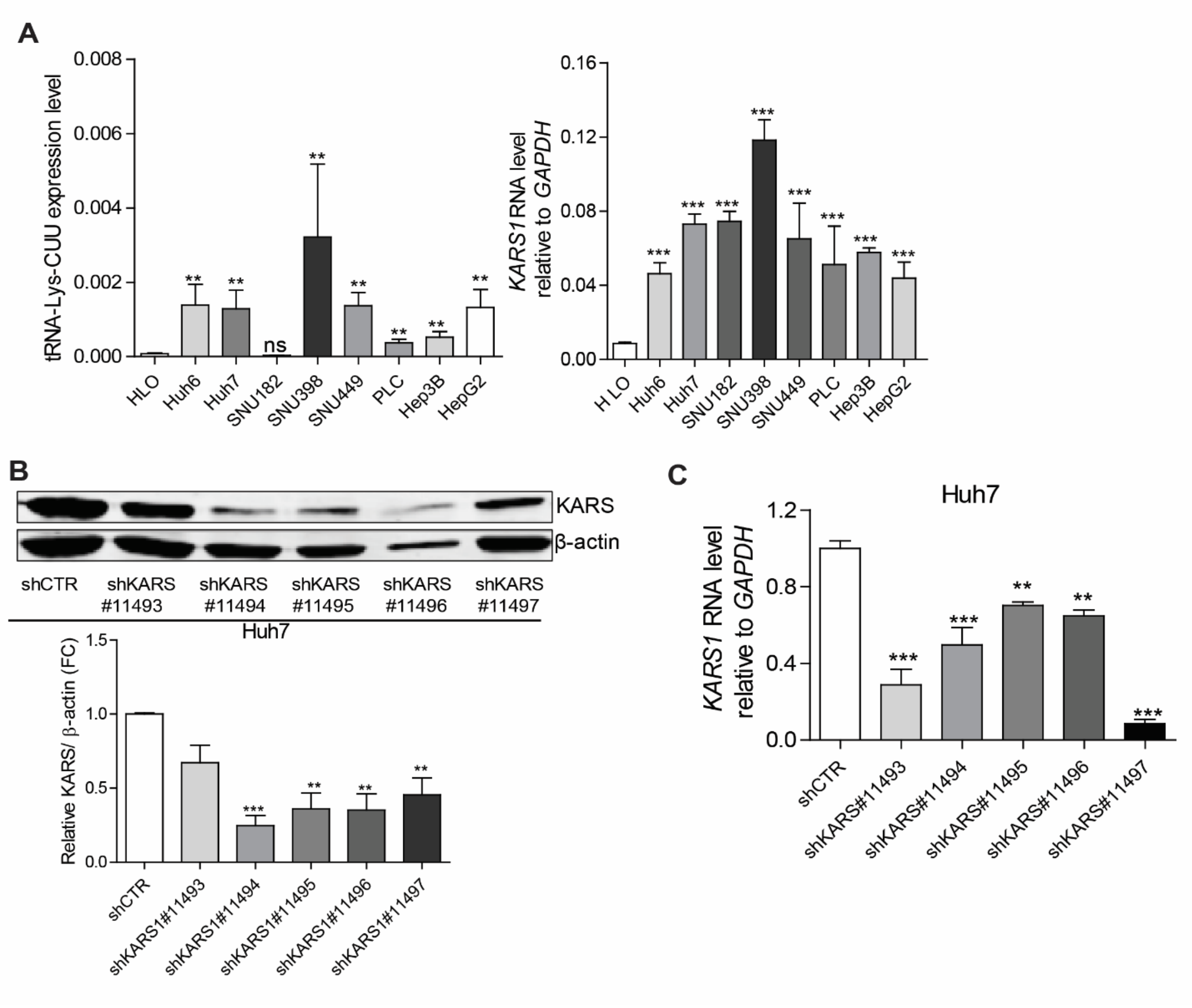

Supplementary Figure 4 The expression level of tRNA-Lys-CUU and *KARS1*, and KARS knockdown efficiency.

(A) The expression level of tRNA-Lys-CUU (left graph) and *KARS1* (right graph) respectively, relative to GAPDH in HCC cell lines compared to normal healthy liver organoids(n = 4-7). (B-C) Knockdown in Huh7 and SNU398 was analyzed in (B) by western blot (upper graph), and the intensity was quantified relative to β-actin (lower graph) by image studio software. In (C) the expression of *KARS1* mRNA upon knockdown was determined by qRT-PCR (mean ± SEM, n = 4-9). **P<0.01; ***P<0.001, by Mann-Whitney test. HLO: healthy liver organoids.

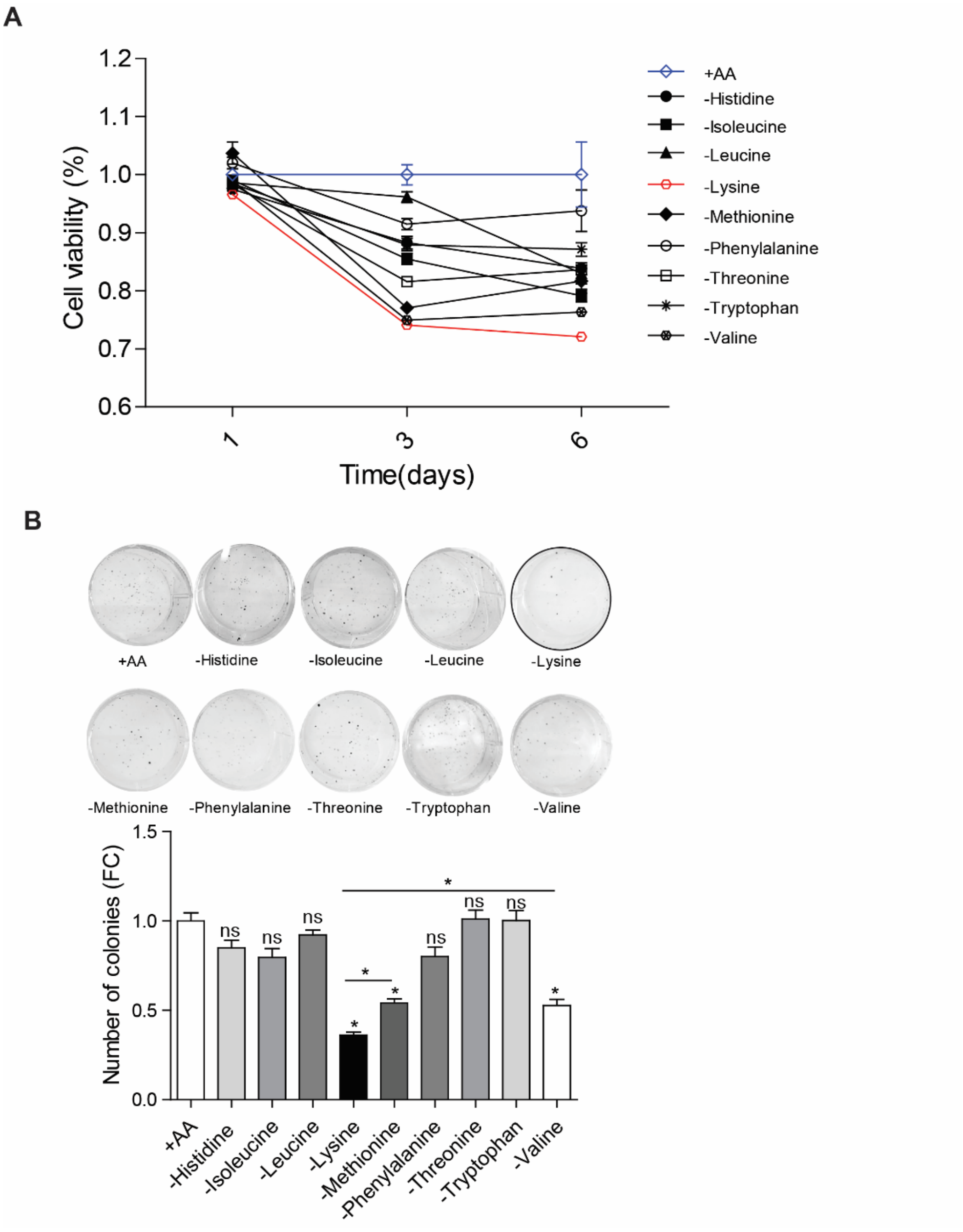

Supplementary Figure 5 Lysine deprivation exerts the strongest inhibitory effects on HCC cell lin growth.(A) The effects of nine essential amino acid deprivation on SNU398 cell growth measured by MTT assay following 1, 3 and 6 days of culture (n = 8). (B) The effects of amino acid deprivation in SNU398 single cell-derived colony formation as assayed 2 weeks after seeding (n = 4). *P<0.05, by the Mann-Whitney test. AA: amino acid; “-” means deprivation.

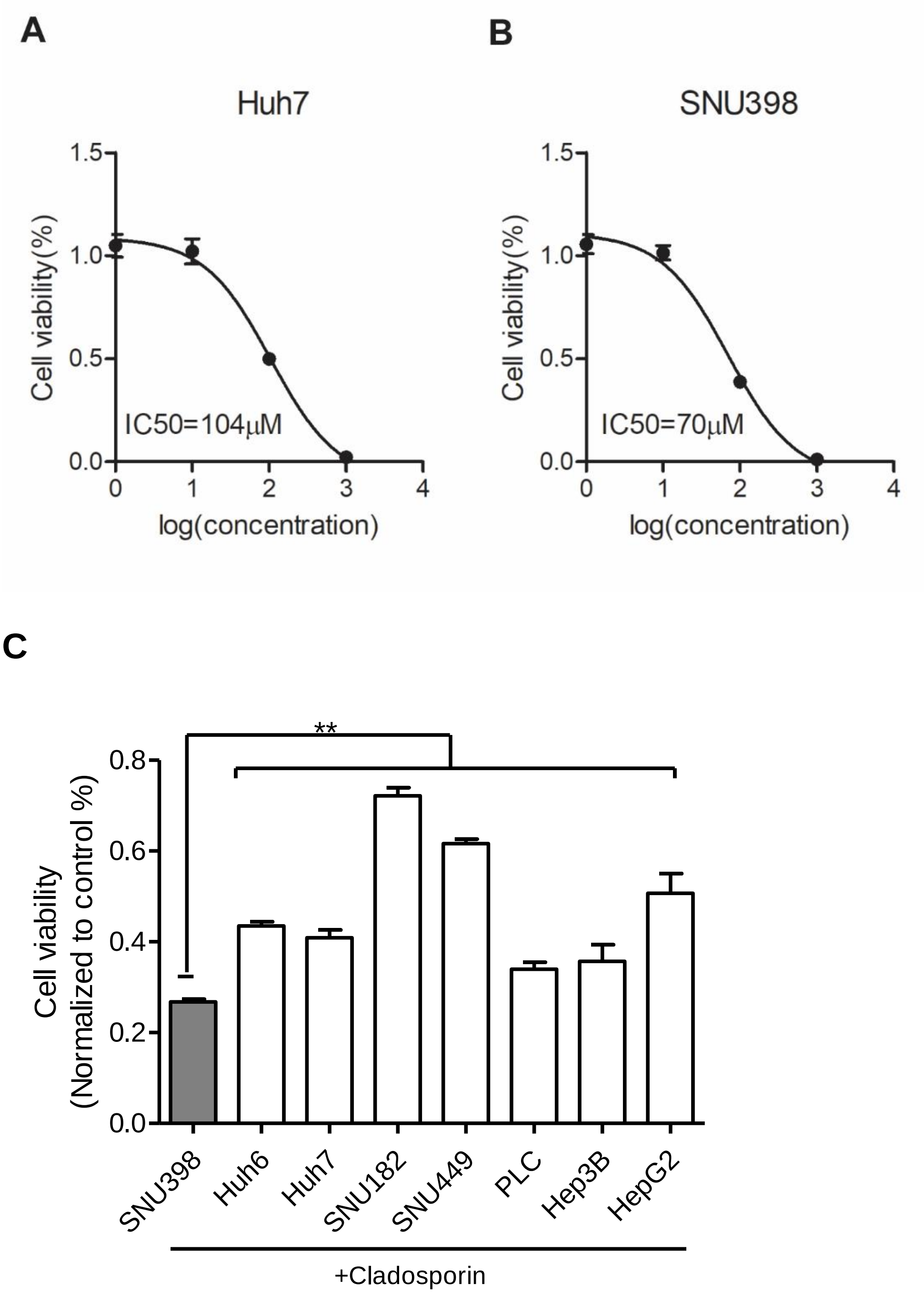

Supplementary Figure 6 The 50% inhibitory concentration of cladosporin was determined by MTT for Huh7 (A) and SNU398 (B) cells following 3 days of treatment. The drug concentrations were 1, 10, 100 and 1000 μM. Data are presented as mean ± SEM.(C) SNU398 cells are more sensitive than other HCC cell lines to treatment of 100 μM cladosporin (n = 6). **P<0.01, by the Mann-Whitney test.

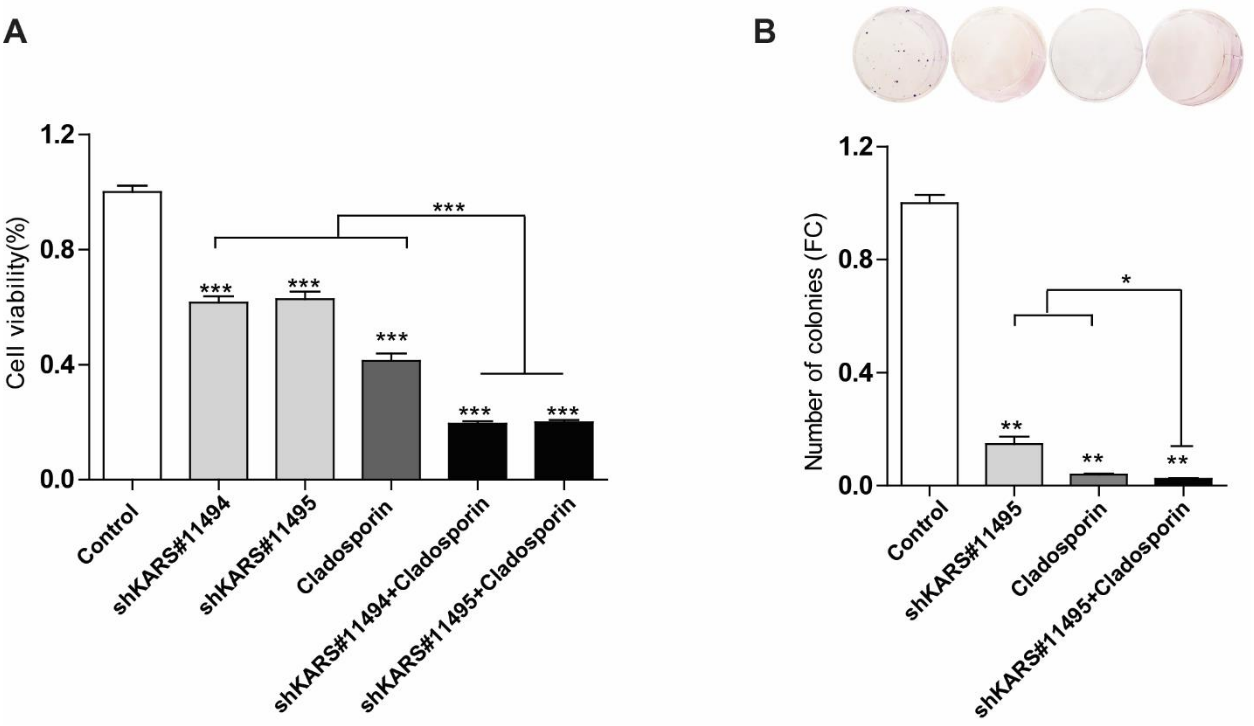

Supplementary Figure 7 The effects of combining cladosporin treatment with KARS silencing in SNU398 cells.

Cell viability (A) and single-colony formation (B) were quantified when combining *KARS1* knockdown and cladosporin treatment (n = 4 -16). *P<0.05; **P<0.01; ***P<0.001, by the Mann-Whitney test.

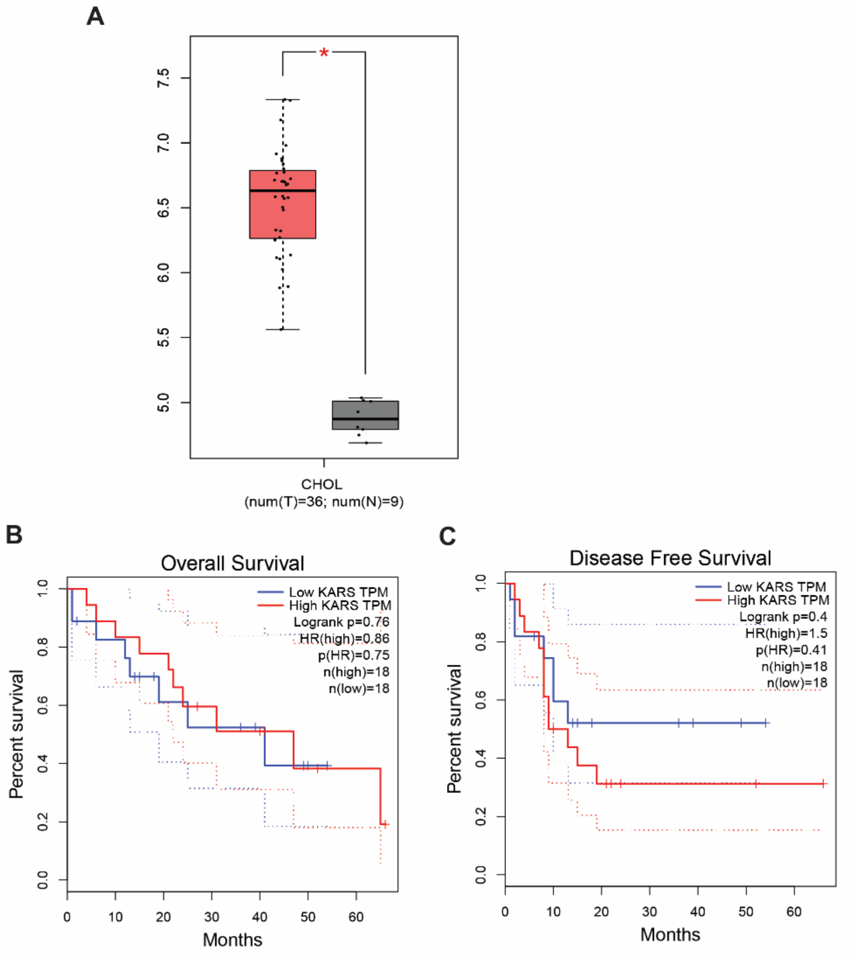

Supplementary Figure 8 The expression level of *KARS1* and association with patient survival in cholangiocarcinoma from the GEPIA database.

(A)The boxplot shows expression level of KARS in CHOL (red color, n = 36). (B) and (C) indicated overall suvival and disease free survival in CHOL, respectivity. *P<0.05; CHOL: cholangiocarcinoma.

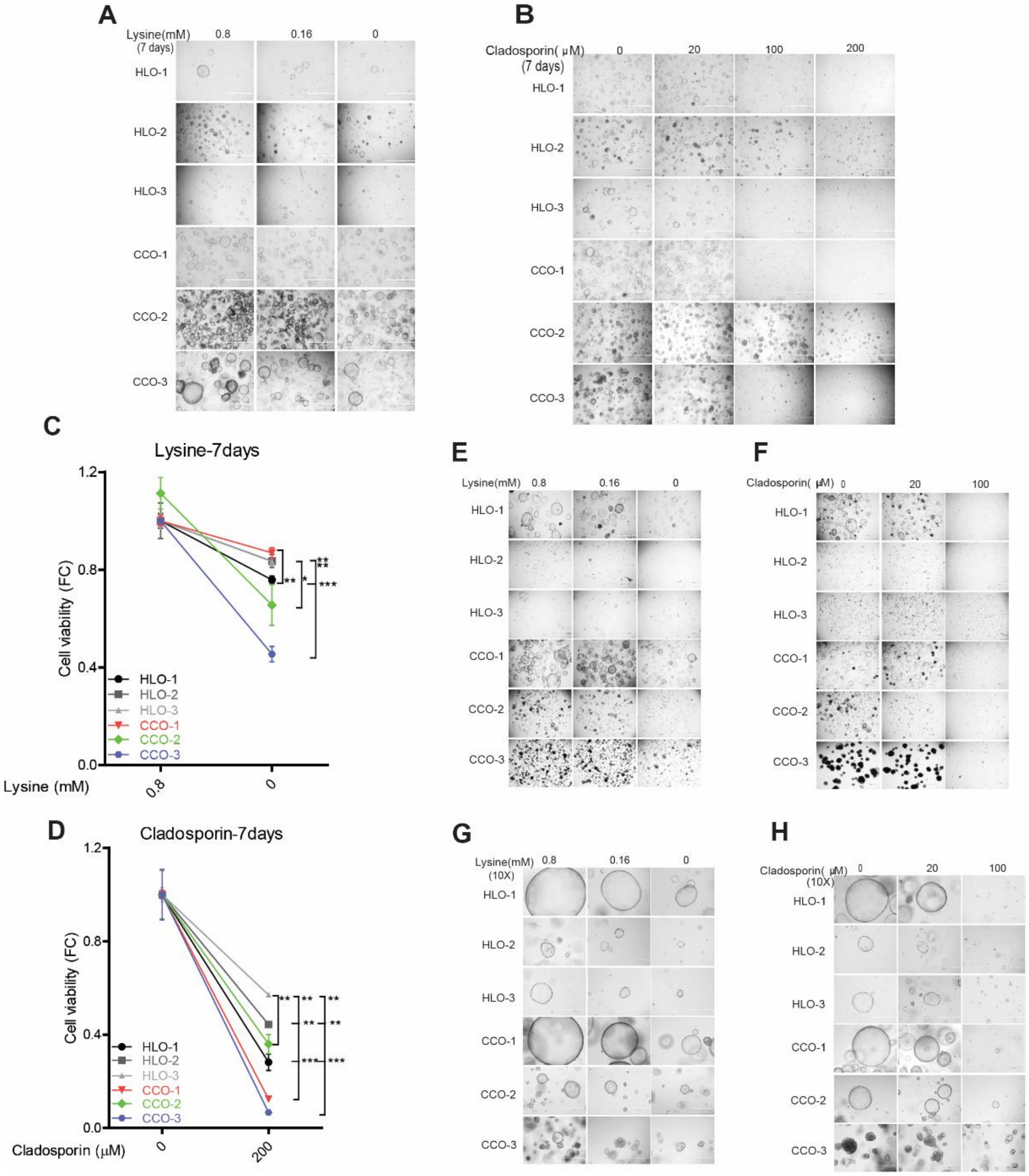

Supplementary Figure 9 Lysine deprivation or treatment with cladosporin affects the growth of patient CC organoids.

(A-B)Images of organoids were treated with different concentrations of lysine (A) or cladosporin (B) for 7 days. (C-D) For cell viability of HLOs and CCOs were treated with lysine deprivation (C) or cladosporin 200 μM (D), high expression of *KARS1,* CCO-3 more sensitive. (E-H) Images were taken after 14 days of culture. (G) and (H) show a 10x magnification highlighting the reduced organoid size after prolonged culture with lysine deprivation or cladosporin. CCO: cholangiocarcinoma organoid; HLO: human liver healthy organoid. *P<0.05； **P<0.01; ***P<0.001, by the Mann-Whitney test.

Supplementary Table 1 Patient characteristics

Characteristic	HCC patients (n=69)	Healthy controls (n=21)
**Age at treatment (years)**		
Mean ± SD	60 ± 16	51 ± 16.2
Median (range)	64 (11-82)	52 (13-88)
**Sex – no. (%)**		
Male	41 (59.4)	12 (57.1)
Female	28 (40.6)	9 (42.9)
**Race – no. (%)**		
White	58 (84.1)	-
African	6 (8.7)	-
Asian	4 (5.8)	-
Not reported	1 (1.4)	21 (100)
**Etiology – no. (%)**		
No known liver disease	20 (29.0)	NA
Alcohol	16 (23.2)	NA
NASH	8 (11.6)	NA
Hepatitis B	8 (11.6)	NA
Hepatitis C + Alcohol	5 (7.2)	NA
Hepatitis B + Alc/HepC/HepD/NASH	4 (5.8)	NA
Hepatitis C	4 (5.8)	NA
Fibrolamellar HCC	3 (4.4)	NA
Other	1 (1.5)	NA
**Hepatitis status – no. (%)**		
Hepatitis B or C positive	21 (30.4)	0 (0)
Chronic Hepatitis B	12 (17.4)	0 (0)
Chronic Hepatitis C	10 (14.5)	0 (0)
**Cirrhosis – no. (%)**		
Yes	50 (72.5)	0 (0)
No	19 (27.5)	21 (100)
**Differentiation grade – no. (%)**		
Good	8 (11.6)	NA
Moderate	35 (50.7)	NA
Poor	14 (20.3)	NA
Unknown	12 (17.4)	NA
**Number of lesions – no. (%)**		
1	38 (55.1)	NA
>1	31 (44.9)	NA
Median (range)	1 (1-11)	NA
**Size of largest lesion (cm)**		
Mean ± SD	7.5 ± 5.1	NA
Median (range)	6.2 (1-24)	NA
**AFP level before resection (ug/l)**		
Mean ± SD	46679.2 ± 375310.5	NA
Median (range)	9 (1-3118700)	NA

Supplementary Table 2 shRNA target sequences

id	symbol	targetTaxonId	targetSeq	geneDesc	oligoSeq	NCBI_geneId	cloneName	vectorId
11493	KARS	human	CGGCGAATCAACATGGTAGAA	lysyl-tRNA synthetase	CCGGCGGCGAATCAACATGGTAGAACTCGA<br>GTTCTACCATGTTGATTCGCCGTTTTTG	3735	NM_005548.1-1214s1c1	pLKO.1
11494	KARS	human	GCCTTTCATCACTTATCACAA	lysyl-tRNA synthetase	CCGGGCCTTTCATCACTTATCACAACTCGAG<br>TTGTGATAAGTGATGAAAGGCTTTTTG	3735	NM_005548.1-883s1c1	pLKO.1
11495	KARS	human	CCTGGAAGTGACTTGCATCAA	lysyl-tRNA synthetase	CCGGCCTGGAAGTGACTTGCATCAACTCGA<br>GTTGATGCAAGTCACTTCCAGGTTTTTG	3735	NM_005548.1-1393s1c1	pLKO.1
11496	KARS	human	CGTGGACCCAAATCAATACTA	lysyl-tRNA synthetase	CCGGCGTGGACCCAAATCAATACTACTCGA<br>GTAGTATTGATTTGGGTCCACGTTTTTG	3735	NM_005548.1-250s1c1	pLKO.1
11497	KARS	human	CCAGAGATACTTGGACTTGAT	lysyl-tRNA synthetase	CCGGCCAGAGATACTTGGACTTGATCTCGA<br>GATCAAGTCCAAGTATCTCTGGTTTTTG	3735	NM_005548.1-724s1c1	pLKO.1

Supplementary Table 3 Primer sequences

Genes	Forward primers (5'-3')	Reverse primer (5'-3')
KARS1	GACGCACAATCCTGAGTTCACC	AGGTGACCTTGTAACTGCCTGTA
GUSB	CAGGTGATGGAAGAAGTGG	GTTGCTCACAAAGGTCACAG
HPRT1	GCTATAAATTCTTTGCTGACCTGCTG	AATTACTTTTATGTCCCCTGTTGACTGG
PMM1	CGAGTTCTCCGAACTGGAC	CTGTTTTCAGGGCTTCCAC
GAPDH	GTCTCCTCTGACTTCAACAGCG	ACCACCCTGTTGCTGTAGCCAA

Supplementary Table 4 List of tRNA expression comparison

Differences of tRNA expression between tumor and TFL patient tissues (n=69) were analyzed using the Wilcoxon matched pairs test. *P<0.05, **P<0.01, ***P<0.001. TFL:tumor-free liver. Red: signficant up-regulation in tumor; Green: signficant down-regulation in tumor; Black: no signficant difference.

Gene	Mean		Wilcoxon matched pairs test
	TFL	Tumor	
tRNA-Leu-CAA	1.522	1.277	***
tRNA-Leu-CAG	5.106	3.551	***
tRNA-Ile-AAU	1.37	1.254	***
tRNA-Ile-GAU	1.076	0.8677	***
tRNA-iMet-CAU	13.49	12.49	***
tRNA-Met-CAU	6.762	5.325	***
tRNA-Stop-UUA	5.32	4.172	***
tRNA-His-GUG	16.01	13.99	***
tRNA-Phe-GAA	1.095	0.801	***
tRNA-Val-AAC	1.8	0.6382	***
tRNA-Val-CAC	2.248	0.7724	***
tRNA-Pro-GGG	0.6167	0.4805	***
tRNA-Pro-CGG	4.063	3.23	***
tRNA-Thr-AGU	0.748	0.5531	***
tRNA-Thr-UGU	2.64	1.922	***
tRNA-Thr-CGU	1.107	0.7374	***
tRNA-Ala-CGC	10.14	4.745	***
tRNA-Ala-GGC	3.448	1.932	***
tRNA-Ala-UGC	8.042	4.49	***
tRNA-Ala-AGC	1.779	1.098	***
tRNA-Tyr-AUA	1.429	1.227	***
tRNA-Tyr-GUA	5.609	2.763	***
tRNA-Stop-CUA	5.562	2.651	***
tRNA-Asn-AUU	2.535	1.669	***
tRNA-Lys-UUU	18.79	9.738	***
**tRNA-Lys-CUU**	**6.867**	**3.389**	*******
tRNA-Asp-AUC	4.942	2.412	***
tRNA-Asp-GUC	0.8559	0.6074	***
tRNA-Glu-UUC	0.9868	0.753	***
tRNA-Cys-GCA	4.178	3.506	***
tRNA-Arg-ACG	0.8352	0.6351	***
tRNA-Arg-CCG	2.109	1.783	***
tRNA-Ser-ACU	1.497	1.283	***
tRNA-seCys-UCA	3.942	3.471	***
tRNA-Val-UAC	1.783	0.4871	**
tRNA-Ser-CGA	5.424	5.389	**
tRNA-Pro-AGG	1.317	1.246	**
tRNA-Gln-CUG	18.07	12.53	**
tRNA-Cys-ACA	0.8275	0.4544	**
tRNA-Arg-UCG	0.682	0.5212	**
tRNA-Ile-UAU	2.046	1.529	ns
tRNA-Gln-UUG	18.85	14.06	ns
tRNA-Asn-GUU	0.4828	0.3918	ns
tRNA-Arg-UCU	0.7006	0.6279	ns
tRNA-Gly-CCC	0.4193	0.3827	ns

Gene	Mean		Wilcoxon matched pairs test
	TFL	Tumor	
tRNA-Gly-GCC	197.2	603.3	***
tRNA-Ser-AGA	2.547	2.894	***
tRNA-Leu-AAG	6.162	8.093	**
tRNA-Leu-UAG	8.082	8.548	**
tRNA-Pro-UGG	3.571	3.923	**
tRNA-Trp-CCA	4.311	5.186	**
tRNA-Glu-CUC	2.303	3.733	*
tRNA-Gly-UCC	1.371	3.731	*
tRNA-Leu-UAA	4.735	5.492	ns
tRNA-Ser-UGA	4.997	5.848	ns
tRNA-Ser-GCU	3.642	4.028	ns
tRNA-Arg-CCU	1.474	1.573	ns
